# Construction and Performance Characterization of BiTmFeSbO_7_/BiTmO_3_ Heterojunction Photocatalyst and the Photocatalytic Degradation of Sulfathiazole Under Visible Light Irradiation

**DOI:** 10.3390/nano15231756

**Published:** 2025-11-23

**Authors:** Jingfei Luan, Xiqi Gou, Ye Yao, Liang Hao, Minghe Ma

**Affiliations:** 1 School of Physics, Changchun Normal University, Changchun 130032, China; 15981009457@139.com (X.G.); yaoye1109@mails.jlu.edu.cn (Y.Y.); 19845486007@139.com (L.H.); 13251704137@139.com (M.M.); 2 State Key Laboratory of Pollution Control and Resource Reuse, School of the Environment, Nanjing University, Nanjing 210093, China

**Keywords:** BiTmFeSbO_7_/BiTmO_3_ heterojunction photocatalyst, Z-scheme, sulfathiazole, visible light irradiation, photocatalytic activity, degradation mechanism

## Abstract

In this study, a novel photocatalytic nanomaterial BiTmFeSbO_7_ was successfully synthesized for the first time by using the solvothermal method. On account of the effective Z-scheme mechanism, the BiTmFeSbO_7_/BiTmO_3_ heterojunction photocatalyst (BTBTHP) could effectively separate the photoinduced electrons and the photoinduced holes, concurrently, the high oxidation potential and reduction potential of the BiTmFeSbO_7_ and the BiTmO_3_ were retained. Additionally, a Z-scheme BTBTHP was synthesized by using an ultrasound-assisted solvothermal approach. As a result, the BTBTHP exhibited excellent photocatalytic performance during the degradation process of the sulfathiazole (STZ). The morphological features, composition distribution, photochemistry properties and photoelectric properties of the prepared samples were investigated by using the comprehensive characterization techniques. Under the condition of visible light irradiation, the BTBTHP demonstrated an excellent removal efficiency of 99.50% for degrading the STZ. Contrastive analysis results indicated that the removal efficiency of the STZ by using the BTBTHP was substantially higher than that by using the BiTmFeSbO_7_, the BiTmO_3_, and the N-doped TiO_2_. The removal rate of the STZ by using the BTBTHP was 1.14 times that by using the BiTmFeSbO_7_, 1.28 times that by using the BiTmO_3_, and 2.71 times that by using the N-doped TiO_2_. Moreover, the stability and the reusability of the BTBTHP were verified through five successive photocatalytic cyclic degradation experiments, indicating that the BTBTHP owned potential for the practical application. The active species which was produced by the BTBTHP were identified as hydroxyl radicals (•OH), superoxide anions (•O_2_^−^), and photoinduced holes (h^+^) by capturing radicals experiments and electron paramagnetic resonance testing experiments. Therefore, the degradation mechanism and the pathway of the STZ could be more comprehensively elucidated. In summary, this study lays a solid foundation for the development and further research of high efficient Z-scheme heterojunction photocatalysts and offers novel insights into sustainable remediation strategies for the STZ pollution.

## 1. Introduction

Antibiotics are a class of compounds which are produced by microorganisms or synthesized artificially through chemical methods [1]. Antibiotics are used to treat microbial infectious diseases and are widely applied in human healthcare, agriculture, animal husbandry, and aquaculture [2,3]. Sulfonamide antibiotics are prolonged synthetic antibiotics which have been utilized for a long time in history. Sulfonamide antibiotics possess broad-spectrum antibacterial activity, significant therapeutic effects, stable properties, and low cost characteristic; thus, sulfonamide antibiotics have become one of the most extensively applied antibiotics [4]. The sulfathiazole (STZ) is one of the most extensively applied sulfonamide antibiotics and has a high detection rate within the aquatic environments. Notably, the STZ cannot be completely metabolized and absorbed within the human and animal bodies. The unabsorbed STZ enters the environment via wastewater discharge in the form of the parent drug or its metabolites, leading to water resource pollution [5]. Numerous studies indicated that the STZ possessed high stability, biological toxicity, water solubility, and strong migration capability [6]. The high mobility of the STZ facilitates its extensive dispersion through water circulation systems; as a result, the STZ is frequently detected in surface waters [7,8] with a concentration of μg/L level [9,10]. The STZ resists the effective removal by using the conventional wastewater treatment systems due to the anionic character and difficult biodegradation characteristic [11]. The accumulating burden of the STZ progressively heightens the potential risks to the organism, including the acute toxicity and the chronic toxicity; concurrently, the microbiologic population undergoes serious threat [12]. The prolonged existence of the STZ which is obtained in the environment facilitates the STZ for going into the food chain; ultimately, human health and animal health are affected [13]. Therefore, developing an efficient and clean method for removing the residual STZ from water bodies is an urgent problem which should be solved.

Up to now, a variety of traditional treatment methods for the STZ removal have been developed and applied, including membrane treatment, biological treatment, and adsorption treatment. However, these methods all exhibit inherent limitations. In recent years, photocatalytic technology has achieved significant attention due to the advantages such as high efficiency, low operational costs, pollution-free operation, high mineralization rate, and environmental friendliness; thus, photocatalytic technology has been widely applied in the environmental field and energy field [14]. As a new advanced oxidation technology, photocatalytic technology has emerged as an effective method for degrading the target pollutants which derive from wastewater [15,16]. Under the condition of the sunlight irradiation, the semiconductor photocatalysts absorb photons for generating the photoinduced electrons and the photoinduced holes (PEPHs). The photoinduced holes oxidize water or hydroxide ions, and thereby the hydroxyl radicals are formed. Meanwhile, the photoinduced electrons reduce the dissolved oxygen which exists in water, and eventually the superoxide anions are formed. The hydroxyl radicals (•OH) and the superoxide anions (•O_2_^−^) are highly oxidative active radicals, which can effectively oxidize and remove the organic pollutants which are contained in wastewater [17].

As a typical photocatalyst, TiO_2_ exhibits responsive properties only under the condition of ultraviolet light irradiation due to the wide band gap energy of 3.2 eV for TiO_2_. However, the ultraviolet light merely accounts for 4% of the solar spectrum energy; thus, the practical applications of TiO_2_ are significantly restricted [18]. In order to more effectively utilize the visible light energy which accounts for 43% of the solar spectrum energy, it is essential to construct composite photocatalysts which are capable of achieving efficient light absorption. In comparison with single metal oxide catalysts, the composite photocatalysts exhibit superior visible light responsive characteristics and superior photocatalytic performance [19,20,21]. According to the existing reports, the A_2_B_2_O_7_-type compounds and the ABO_3_-type compounds exhibit excellent photocatalytic performance under the condition of visible light irradiation (VLIRN). For instance, Liu et al. utilized the Bi_2_Zr_2_O_7_ nanocatalyst under the condition of VLIRN; as a result, the photocatalytic removal efficiency of tetracycline was as high as 81.3% [22]. Researchers who came from Tamil Nadu indicated that the Sm_2_Ti_2_O_7_ photocatalyst could efficiently degrade rhodamine B dye within wastewater under the condition of VLIRN [23]. Girish et al. reported that the removal rate of ofloxacin by using the BiFeO_3_ photocatalyst reached 80% after VLIRN of 180 min [24]. Thirumalairajan et al. reported that the degradation removal rate of rhodamine B and methylene blue by using the LaFeO_3_ nanocatalyst reached 90.66% and 92.85%, respectively, under the condition of VLIRN [25].

In previous studies, we extensively explored the potential for enhancing the photocatalytic performance of the Bi_2_InNbO_7_ photocatalyst through structural modification. The Bi_2_InNbO_7_ was a visible light responsive photocatalyst which possessed a stable pyrochlore structure [26]. We also drew inspiration from previous experiments on how to improve the photocatalytic activity of the TiO_2_ photocatalyst by doping elements into the TiO_2_ photocatalyst. Cheng et al. synthesized the Fe-doped TiO_2_; as a result, the Fe-doped TiO_2_ showed significantly enhanced photocatalytic degradation efficiency of terramycin compared with pure TiO_2_ [27]. Desire et al. synthesized the Tm-doped TiO_2_; obviously, the results demonstrated that the Tm-doped TiO_2_ had a higher photocatalytic degradation efficiency of methylene blue compared with the undoped TiO_2_ [28]. Chen et al. synthesized the Sb-doped TiO_2_; as a result, the Sb-doped TiO_2_ exhibited significantly enhanced photocatalytic degradation efficiency of methyl orange in comparison with pure TiO_2_ [29]. Zhang et al. prepared the Bi-doped TiO_2_; obviously, the results indicated that the Bi-doped TiO_2_ possessed enhanced photocatalytic degradation efficiency of phenol compared with the undoped TiO_2_ [30]. In conclusion, the abovementioned studies all demonstrate that the addition of the elements such as Fe, Tm, Sb, and Bi can boost the photocatalytic activity of TiO_2_. Based on the analysis of the above experimental results, we can replace certain specific elements in Bi_2_InNbO_7_ for increasing the carrier concentration; thereby, the photocatalytic activity of the doped Bi_2_InNbO_7_ photocatalyst is higher than that of the Bi_2_InNbO_7_ photocatalyst. In summary, for the Bi_2_InNbO_7_ photocatalyst, we, respectively, utilize Tm^3+^, Fe^3+^, and Sb^5+^ for replacing Bi^3+^, In^3+^, and Nb^5+^; as a result, the new catalyst BiTmFeSbO_7_ which possesses enhanced photocatalytic activity is synthesized. Similarly, in accordance with the existing literature, the BiTbO_3_ had potential photocatalytic activity in the visible light region [31]; subsequently, we used Tm^3+^ to replace Tb^3+^ which belonged to BiTbO_3_ for synthesizing the new catalyst BiTmO_3_ which owned enhanced photocatalytic activity. Therefore, the novel photocatalysts BiTmFeSbO_7_ and BiTmO_3_ which are designed and synthesized in this paper possess superior photocatalytic activity.

However, the single photocatalyst exhibits a severe recombination phenomenon of the PEPH. In order to address the above issue, the heterojunction photocatalysts have been proposed. The formation of the heterojunctions can increase the separation rate of the PEPH [32], and thereby the photocatalytic activity of the heterojunction photocatalysts may be improved. In comparison with the traditional type-II heterojunction photocatalysts, the Z-scheme heterojunction photocatalysts have attracted more attention due to their superior photocatalytic activity; concurrently, the Z-scheme heterojunction photocatalysts have become the current mainstream heterojunctions [33,34].

The design of the Z-scheme heterojunctions draws inspiration from natural photosynthesis which occurs in the plants. The Z-scheme heterojunction is a type of heterojunction which is composed of distinct reduction centers and oxidation centers which derive from two or more semiconductors. Upon absorbing light energy equal to or greater than their respective band gap energy, two distinct systems are formed [35]. Different from the photocatalytic degradation mechanism of organic pollutants by using the traditional composite catalysts, the Z-scheme heterojunction structure enables the photogenerated holes to remain in the valence band of the Z-scheme heterojunction catalyst with a more positive electrochemical potential; simultaneously, the photogenerated electrons are allowed to stay in the conduction band of the Z-scheme heterojunction catalyst with a more negative electrochemical potential. Therefore, the photogenerated electrons possess a stronger reducing ability, and, concurrently, the photogenerated holes possess a stronger oxidizing ability [36,37,38]. Thus far, the Z-scheme heterojunction photocatalysts have made significant progress in the photocatalytic area. For instance, Mafa et al. successfully fabricated a Z-scheme ZnMoO_4_/BiFeWO_6_/rGO ternary composite heterojunction photocatalyst; obviously, the results showed that the ZnMoO_4_/BiFeWO_6_/rGO ternary composite heterojunction photocatalyst achieved a removal rate of 98% for degrading the anthraquinone blue 25 after VLIRN of 180 min [39]. Liu et al. successfully synthesized a direct Z-scheme BiOBr/CuI heterojunction photocatalyst; as a result, the BiOBr/CuI heterojunction photocatalyst possessed a high photocatalytic activity for the degradation of the phenol under the condition of VLIRN [40]. In accordance with the analysis of the above experimental results, the construction of the Z-scheme heterojunctions can significantly enhance the photocatalytic activity of the photocatalyst.

According to the above insights, a suitable energy band structure should be designed; thus, we propose a novel direct Z-scheme BiTmFeSbO_7_/BiTmO_3_ heterojunction photocatalyst (BTBTHP) for the efficient degradation of the STZ under the condition of VLIRN. The innovative research content of this study is the successful preparation of new visible light responsive BiTmFeSbO_7_ photocatalysts, the BiTmO_3_ photocatalyst and the BTBTHP, by using the solvothermal method. This study investigates the photocatalytic degradation efficiency of the STZ by using the BTBTHP, the BiTmFeSbO_7_ photocatalyst, the BiTmO_3_ photocatalyst, and the nitrogen-doped TiO_2_ (N-T) under the condition of VLIRN. The photocatalytic activity of the BTBTHP, the BiTmFeSbO_7_ photocatalyst, the BiTmO_3_ photocatalyst, and the N-T photocatalyst are evaluated. The results showed that the BTBTHP exhibited excellent photocatalytic activity compared with the BiTmFeSbO_7_ photocatalyst, the BiTmO_3_ photocatalyst and the nitrogen-doped TiO_2_ (N-T); meanwhile, the reusability and the stability of the BTBTHP are verified by accomplishing the cyclic experiments. The crystal structure, morphological characteristics, and optical properties of the BTBTHP are thoroughly investigated; concurrently, the possible direct Z-scheme mechanism of the BTBTHP is verified. This study systematically explores the types of radicals, the reaction mechanisms, and the degradation pathways during the photocatalytic degradation process of the STZ. This research provides important process parameters and a theoretical basis for developing an environmentally friendly photocatalytic reaction system which can degrade the STZ efficiently in pharmaceutical wastewater.

## 2. Experimental Section

### 2.1. Materials and Reagents

Bi(NO_3_)_3_·5H_2_O (purity 99.0%), Fe(NO_3_)_3_·9H_2_O (purity 99.9%), and NaSbO_3_ (purity 98.0%) were procured from Aladdin Industrial Corporation, Shanghai, China. Tm(NO_3_)_3_·5H_2_O (purity 99.9%) was purchased from Merck, Shanghai, China. Ethylenediaminetetraacetic acid (EDTA, C_10_H_16_N_2_O_8_, purity 99.99%) and benzoquinone (BQ, C_6_H_4_O_2_, purity 99.0%) were obtained from Shanghai Macklin Biochemical Co., Ltd. (Shanghai, China). Absolute ethanol (C_2_H_5_OH, purity 99.5%), isopropyl alcohol (IPA, C_3_H_8_O, purity 99.999%), and the pollutant STZ (C_9_H_9_N_3_O_2_S_2_, purity 99.0%) were all sourced from Aladdin Industrial Corporation, Shanghai, China.

### 2.2. Preparation Method of BiTmO_3_

In this study, the BiTmO_3_ photocatalyst was synthesized employing the solvothermal method. In the first instance, isovolumetric precursor solutions of Bi(NO_3_)_3_·5H_2_O (0.21 mol/L) and Tm(NO_3_)_3_·5H_2_O (0.21 mol/L) were thoroughly mixed and magnetically stirred for 1250 min. Subsequently, the obtained homogeneous precursor mixture was transferred into an autoclave which was furnished with polytetrafluoroethylene at a temperature of 230 °C for 750 min. Then, under the condition of a nitrogen atmosphere, the mixture was heated in a tube furnace at a heating rate of 5 °C/min until the temperature reached 1170 °C, and then the mixture was maintained at this temperature for 1620 min. This procedure effectively produced pure BiTmO_3_ powder.

### 2.3. Preparation Method of BiTmFeSbO_7_

The BiTmFeSbO_7_ photocatalyst was also synthesized by using the solvothermal technique. The isovolumetric precursor solutions of Bi(NO_3_)_3_·5H_2_O (0.21 mol/L), Tm(NO_3_)_3_·5H_2_O (0.21 mol/L), Fe(NO_3_)_3_·9H_2_O (0.21 mol/L), and NaSbO_3_ (0.21 mol/L) were mixed. After being thoroughly homogenized and magnetically stirred for 1500 min, the precursor solution mixture was transferred into an autoclave which was lined with polytetrafluoroethylene, heated to 210 °C, and maintained at this temperature for 1250 min. The mixture was then heated in a tube furnace at a heating rate of 8 °C/min until a final temperature of 1180 °C was reached; subsequently, the above mixture was maintained at the temperature of 1180 °C for 1560 min. The pure BiTmFeSbO_7_ powder was successfully synthesized by the above method.

### 2.4. Synthesis of Nitrogen-Doped TiO_2_

The complete production process of the nitrogen-doped TiO_2_ photocatalyst is provided in the Supplementary Materials (Section S1).

### 2.5. Optimal Ratio of BiTmFeSbO_7_/BiTmO_3_ Heterojunction Photocatalyst

The molar ratios of the BiTmFeSbO_7_ to the BiTmO_3_ were controlled at 1:3, 1:2, 1:1, 2:1, and 3:1; subsequently, the corresponding composite catalyst samples were denoted as BTBTHP-(1:3), BTBTHP-(1:2), BTBTHP, BTBTHP-(2:1), and BTBTHP-(3:1), respectively. Table S1 displays the removal rates of the STZ by using the BTBTHP which possesses different proportions during the photocatalytic degradation process of the STZ under the condition of VLIRN. As summarized in Table S1, the experiments for degrading the STZ under the condition of VLIRN yielded the following results: when the BTBTHP which possessed the molar ratio of 1:1 for the BiTmFeSbO_7_ to the BiTmO_3_ was utilized for degrading the STZ, the degradation efficiency of the STZ reached 99.5%, whereas the removal rates of the STZ by using other composite catalysts which owned other molar ratios of the BiTmFeSbO_7_ to the BiTmO_3_ were below 90%. The above results indicated that the heterojunction photocatalyst which was prepared with a molar ratio of 1:1 for the BiTmFeSbO_7_ to the BiTmO_3_ facilitated the formation of the largest heterojunction catalyst interface, and thereby the separation efficiency of the PEPH was accelerated; ultimately, the strongest photocatalytic activity of the BTBTHP was realized. When the molar ratio of the BiTmFeSbO_7_ to the BiTmO_3_ is less than 1, the formed heterojunction catalyst interface is relatively small; as a result, the weak photocatalytic activity of the heterojunction catalyst was realized. Conversely, when the molar ratio of the BiTmFeSbO_7_ to the BiTmO_3_ for the heterojunction catalyst exceeds 1, the excessive nanoparticles occupy the active sites which derive from the surface of the heterojunction catalyst; similarly, a relatively low removal rate of the STZ was obtained during the photocatalytic degradation process of the STZ by using the heterojunction catalyst which owned a molar ratio of 2:1 or 3:1 for the BiTmFeSbO_7_ to the BiTmO_3_. In summary, the optimal molar ratio of BiTmFeSbO_7_ to the BiTmO_3_ for producing the BTBTHP is 1:1. The heterojunction composite catalyst BTBTHP which was formed in accordance with a molar ratio of 1:1 for BiTmFeSbO_7_ to the BiTmO_3_ was utilized in both the photocatalytic performance characterization and the STZ degradation experiments throughout this study.

### 2.6. Synthesis of BiTmFeSbO_7_/BiTmO_3_ Heterojunction Photocatalyst

In this study, the ultrasonic-assisted solvothermal method was used to synthesize BTBTHP. Initially, equal mole numbers of the BiTmO_3_ photocatalyst and the BiTmFeSbO_7_ photocatalyst which were previously synthesized by using the solvothermal method were mixed with octanol. The mixture was then sonicated in an ultrasonic bath for 60 min. In order to promote the surface binding of BiTmO_3_ nanoparticles and BiTmFeSbO_7_ nanoparticles, the mixture was vigorously stirred at 180 °C for 200 min, and thus the BiTmFeSbO_7_/BiTmO_3_ heterostructural catalyst was formed. The centrifugal separation technique was employed to obtain the product once the centrifugal product had cooled to room temperature. The obtained product was washed with ethanol multiple times to ensure complete purification. As to the subsequent usage, the purified powder was placed in a desiccator after being dried for 360 min at 60 °C in a vacuum oven. Eventually, the BTBTHP was successfully prepared.

Furthermore, in accordance with the above-described preparation method, the estimated costs of the N-T, the BiTmFeSbO_7_, the BiTmO_3_, and the BTBTHP were approximately USD 353.5 per 100 g, USD 54.3 per 100 g, USD 77.8 per 100 g, and USD 63.4 per 100 g, respectively. It was evident that the cost of the N-T was 6.5 times higher than that of the BiTmFeSbO_7_, 4.5 times higher than that of the BiTmO_3_, and 5.6 times higher than that of the BTBTHP.

### 2.7. Characterization

The descriptions of the characterization details are reported in the Supplementary Materials (Section S2).

### 2.8. Photoelectrochemical Experiments

The electrochemical impedance spectroscopy (EIS) experiment was conducted on a CHI660D electrochemical workstation which derived from Chenhua Instrument Co., Ltd., Shanghai, China. The working electrode, counter electrode, and reference electrode constituted the standard three-electrode system of this experiment. In the experiment, the Ag/AgCl electrode was utilized as the reference electrode, and, simultaneously, a platinum plate served as the counter electrode; eventually, the prepared catalyst was used as the working electrode. An aqueous solution of 0.5 mol/L Na_2_SO_4_ with a pH value of 7 was used as the electrolyte. The light source which was utilized during the process of the experiment was composed of a 500 W xenon lamp and a 420 nm cutoff filter.

### 2.9. Experimental Setup and Procedure

A set of 12 quartz tubes was utilized for each experiment, and each quartz tube contained 40 mL of the reaction solution. As to the STZ solution, the total reaction volume was 480 mL. The photodegradation experiments were performed using a dosage of 0.5 g/L nanocatalyst such as BiTmFeSbO_7_, BiTmO_3_, or BTBTHP. The initial concentration of STZ in the pharmaceutical wastewater solution was 0.032mmol/L. The degradation experiments were carried out in a photocatalytic reactor (CEL-LB70, China Educational Jinguang Technology Co., Ltd., Beijing, China). In order to promote the uniform dispersion of the photocatalytic samples throughout the photocatalytic reaction system and reach the adsorption saturation of the STZ, a dark adsorption process which lasted for 45 min was accomplished in darkness.

During the photocatalytic degradation process, the STZ, a xenon lamp (500 W), and a cut-off filter (420 nm) were used to simulate the effect of VLIRN. Five milliliters of the reaction solution samples were taken out for examination at 15 min intervals during the photocatalytic reaction process. Each sample was then centrifuged for 10 min at 7200 rpm to extract the clear liquid, which was used for additional investigation and analysis. The residual concentration of the STZ was determined by using an Agilent 200 high-performance liquid chromatography (HPLC) system (Agilent Technologies, Palo Alto, CA, USA) by injecting a volume of 10 µL into the HPLC at a flow rate of 1 mL/min for analysis.

The mineralization outcomes of the STZ in the reaction solution were analyzed by using a TOC analyzer (TOC-5000 A, Shimadzu Corporation, Kyoto, Japan). Potassium phthalate (KHC_8_H_4_O_4_) was used as the reference substance for measuring the concentration of TOC during the photocatalytic degradation process of the STZ. The potassium phthalate served as the calibration standard. Concurrently, the carbon concentration range was established from 0 mg/L to 100 mg/L.

A calibration study of intermediate products which were produced during the photocatalytic degradation process of the STZ was conducted by using liquid chromatography–mass spectrometry (LC–MS) with a Thermo Quest LCQ Duo system (Thermo Fisher Scientific Corporation, Waltham, MA, USA) and a Beta basic-C18 high-performance liquid chromatography column (Thermo Fisher Scientific Corporation, Waltham, MA, USA). The mobile phase which existed in the LC–MS apparatus consisted of a mixture of 60% methanol and 40% ultrapure water. When the photocatalytic reaction for degrading the STZ stopped, 20 µL of the solution which was generated during the photocatalytic degradation process of the STZ was automatically injected into the LC–MS equipment. In order to identify the intermediate products easily, the range for the mass-to-charge ratio of *m*/*z* was established from 50 to 500.

## 3. Results and Discussion

### 3.1. Characterization of Photocatalysts

#### 3.1.1. Analysis of Morphological and Structural Characterization

**Figure 1:**
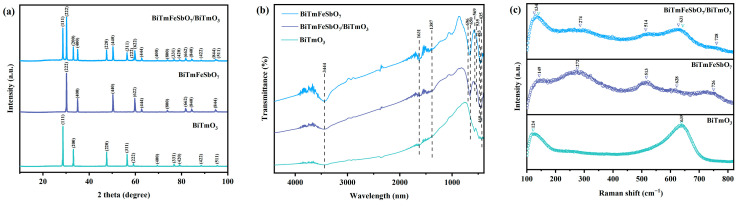
(**a**) The XRD spectra, (**b**) the FTIR spectra, and (**c**) the Raman spectra of the BTBTHP, the BiTmFeSbO_7_ photocatalyst, and the BiTmO_3_ photocatalyst.

Figure 1a presents the XRD patterns of BTBTHP, BiTmFeSbO_7_, and BiTmO_3_. The diffractive peaks and the crystal plane indices which were observed in the XRD pattern of BTBTHP were consistent with that of BiTmFeSbO_7_ and that of BiTmO_3_, confirming that the BTBTHP was successfully synthesized. Figure S1 presents the XRD pattern of the BiTmO_3_ photocatalyst. As shown in Figure S1, the BiTmO_3_ sample corresponded to the JCPDS card number PDF#04-009-5546. Figure S2 displays the XRD pattern of the BiTmFeSbO_7_ photocatalyst. As shown in Figure S2, the BiTmFeSbO_7_ sample corresponded to the JCPDS card number PDF#73-0444. Figure S3a shows the XRD pattern and the Rietveld refinement results of BiTmFeSbO_7_. Figure S3b indicates the atomic structure of BiTmFeSbO_7_. Figure S4a displays the XRD pattern and the Rietveld refinement results of BiTmO_3_. Figure S4b indicates the atomic structure of BiTmO_3_. In order to obtain the photophysical parameters and crystal structure parameters of BiTmFeSbO_7_ and BiTmO_3_, the XRD data of BiTmFeSbO_7_ and BiTmO_3_ were refined by the Rietveld method using the Materials Studio 2020, as shown in Figures S3a and S4a, respectively. In accordance with Figure S3a, the precise results of BiTmFeSbO_7_ which achieved an R_P_ factor of 4.49%, indicated a significant consistency between the experimental intensities of the diffractive peaks for BiTmFeSbO_7_ and the theoretical intensities of the diffractive peaks for BiTmFeSbO_7_. Based on Figure S4a, the precise results of BiTmO_3_ which achieved an R_P_ factor of 6.73% illustrated a significant consistency between the experimental intensities of the diffractive peaks for BiTmO_3_ and the theoretical intensities of the diffractive peaks for BiTmO_3_. These findings confirm that the BiTmFeSbO_7_ photocatalyst possesses the pyrochlore-type structure, while the BiTmO_3_ photocatalyst has the fluorite structure. Furthermore, the BiTmFeSbO_7_ photocatalyst and the BiTmO_3_ photocatalyst exhibit a single pure phase. BiTmFeSbO_7_ crystallized in the cubic crystal system with the space group of Fd3m, while BiTmO_3_ crystallized in the cubic crystal system with the space group of Fm3m. The lattice constant of BiTmFeSbO_7_ was determined to be 10.563 Å; concurrently, the lattice constant of BiTmO_3_ was confirmed to be 5.396 Å. The crystal structures of the BiTmFeSbO_7_ photocatalyst and the BiTmO_3_ photocatalyst were constructed by using the relevant atomic coordinates, crystal systems, lattice constants, structural parameters, and space groups. In addition, Table S2 presents the atomic coordinates and structural parameters of BiTmFeSbO_7_. Table S3 represents the atomic coordinates and structural parameters of the BiTmO_3_ photocatalyst. The above conclusions demonstrate the structural stability of the synthetic BiTmFeSbO_7_ photocatalyst and the BiTmO_3_ photocatalyst. Simultaneously, the BiTmFeSbO_7_ photocatalyst and the BiTmO_3_ photocatalyst highlight the application potential in the water environmental field as efficient photocatalysts.

The unique crystal structure of the BiTmFeSbO_7_ photocatalyst consists of MO_6_ (M = Fe^3+^ and Sb^5+^) octahedra which are interconnected by Bi^3+^ and Tm^3+^. In BiTmFeSbO_7_, there are two types of A-O bonds (A = Bi^3+^ and Tm^3+^), with the longer A–O(1) bond length of 2.487 Å and the shorter A–O(2) bond length of 2.165 Å. The difference in bond length is expected to cause the distortion of MO_6_ octahedra; as a result, the recombination rate of the PEPH decreases [41]. In the crystal structure of BiTmFeSbO_7_, the M-O-M bond angle was measured to be 134.02°. Previous studies had demonstrated that the photocatalytic activity of the photocatalyst increased when the bond angle of the photocatalyst approached 180° [41]. The larger M-O-M bond angle which belongs to the BiTmFeSbO_7_ photocatalyst further enhances the photocatalytic activity of BiTmFeSbO_7_. These findings suggest that the outstanding photocatalytic performance of BiTmFeSbO_7_ can be ascribed to the unique crystal structure of BiTmFeSbO_7_. Meanwhile, the above results also indicate the stability of the sample structure for BiTmFeSbO_7_. Simultaneously, it is emphasized that a thorough understanding of the crystallography for the BiTmFeSbO_7_ photocatalyst is crucial for optimizing the photocatalytic activity of the BiTmFeSbO_7_ photocatalyst.

Figure S5 displays the XRD pattern of the nitrogen-doped titanium dioxide. In accordance with Figure S5, the XRD pattern of the nitrogen-doped titanium dioxide revealed that the sample exhibited characteristic crystal planes of the anatase titanium dioxide (JCPDS No. 21-1272).

In order to analyze the functional groups and chemical bonds which belonged to the BTBTHP, the BiTmFeSbO_7_ photocatalyst, and the BiTmO_3_ photocatalyst, the Fourier transform infrared (FTIR) spectrogram analysis was performed by using the FTIR spectrometer (Beijing North Finray Analytical Instrument Co., Ltd., Beijing, China). Figure 1b displays the FTIR spectra of the BiTmFeSbO_7_ photocatalyst, the BTBTHP, and the BiTmO_3_ photocatalyst. As shown in Figure 1b, the FTIR spectra exhibited several characteristic absorption peaks, which were associated with the Bi-O bond, Tm-O bond, Fe-O bond, Sb-O bond, and Sb-O-Sb bond. The stretching vibration of the Bi-O bond occurred at 435 cm^−1^ [42,43], while the bending vibration of the Tm-O bond was observed at 650 cm^−1^ [44]. The stretching vibration of the Fe-O bond was related to the peak at 483 cm^−1^ or 535 cm^−1^ [45]; nevertheless, the bending vibration of the Sb-O bond was observed at 460 cm^−1^ [46]. The bending vibration of the Sb-O-Sb bond was detected at 569 cm^−1^ and 696 cm^−1^ [47]. A broad band which was centered at 3444 cm^−1^ indicated the stretching vibration mode of the hydroxyl group which was adsorbed by water [48,49]. Equally, the broad band which was detected at 1631 cm^−1^ corresponded to the bending vibration of hydroxyl groups which existed on the surface of the catalyst [50]. Furthermore, the absorption peak which existed at approximately 1387 cm^−1^ was attributed to the OH vibration of water molecules which were adsorbed on the sample surface [51].

The molecular structures of the BiTmO_3_ photocatalyst, the BiTmFeSbO_7_ photocatalyst, and the BTBTHP were investigated by using the Raman spectroscopy for probing their characteristic chemical bond vibrations. Figure 1c displays the Raman spectra of the BiTmFeSbO_7_ photocatalyst, the BiTmO_3_ photocatalyst, and the BTBTHP. In the Raman spectrum of the BiTmFeSbO_7_ photocatalyst, the peak which was observed at 149 cm^−1^ corresponded to the Fg mode and Ag mode of the Tm-O bond [52]. The peak which was observed at 272 cm^−1^ was attributed to the stretching vibration mode of the Bi-O bond [53]. The peak located at 513 cm^−1^ was associated with the A_1g_ bending vibration mode of the O-M-O bond in the MO_6_ (M = Fe^3+^ and Sb^5+^) octahedra [54]. The peak located at 628 cm^−1^ could be attributed to the combined modes of A_1g_ and F_2g_ [54,55]. Additionally, the peak located at 726 cm^−1^ was related to the F_2g_ stretching vibration mode of the M-O bond and the O-M-O bond which existed in the MO_6_ octahedra [54,56]. In the Raman spectrum of the BiTmO_3_ photocatalyst, the peak located at 124 cm^−1^ was attributed to the stretching vibration mode of the Bi-O bond [57], while the peak located at 639 cm^−1^ corresponded to the F_g_ mode and the A_g_ mode of the Tm-O bond [58]. Additionally, the Raman spectrum of the BTBTHP included the different absorption peaks derived from the BiTmFeSbO_7_ photocatalyst and the BiTmO_3_ photocatalyst. Therefore, the BTBTHP possessed peaks which were located at 134 cm^−1^, 274 cm^−1^, 514 cm^−1^, 631 cm^−1^, and 728 cm^−1^. These results provide indirect evidence for the successful preparation of the BiTmO_3_ photocatalyst, the BiTmFeSbO_7_ photocatalyst, and the BTBTHP. Additionally, Figures S6–S8 show the Raman spectra of the BiTmO_3_ photocatalyst, the BiTmFeSbO_7_ photocatalyst, and the BTBTHP, respectively.

**Figure 2:**
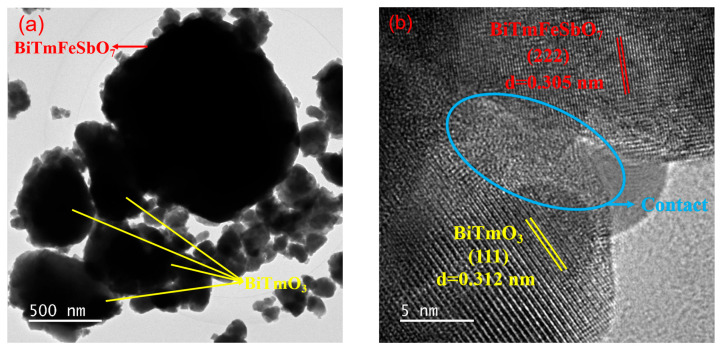
(**a**) The TEM morphology image of the BTBTHP and (**b**) the HRTEM image of the BTBTHP.

**Figure 3:**
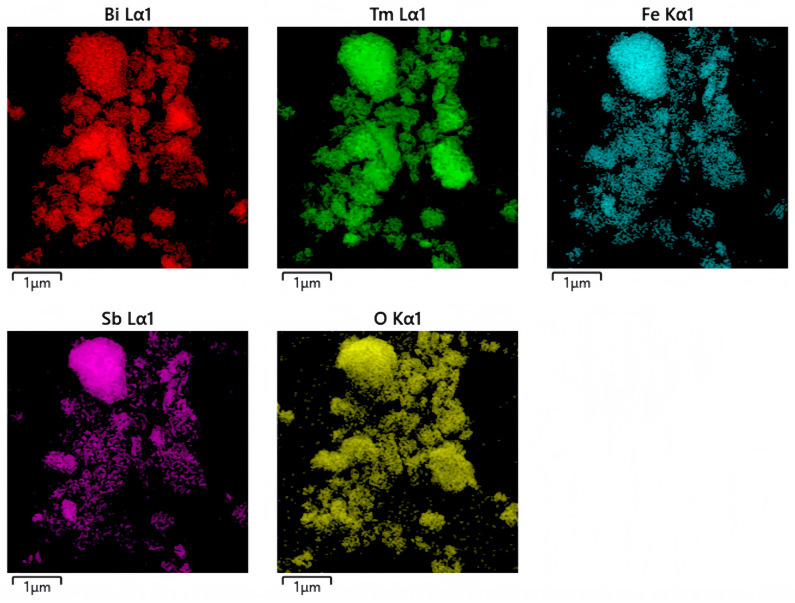
The EDS element scanning mapping of the BTBTHP (Bi, Tm, Fe, Sb, and O from BiTmFeSbO_7_; simultaneously, Bi, Tm, and O from BiTmO_3_).

**Figure 4:**
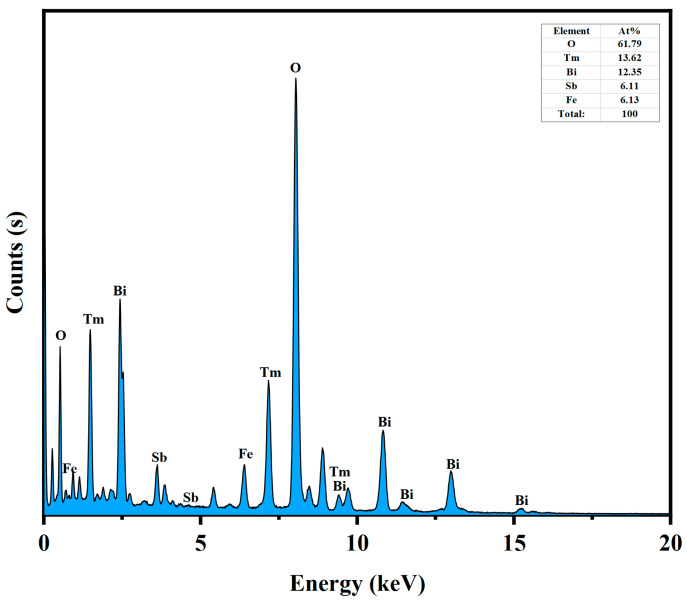
The EDS spectrum of the BTBTHP.

The microstructure of the BTBTHP was investigated by using the transmission electron microscopy (TEM); simultaneously, the elemental composition content and the elemental qualitative judgment of the BTBTHP were determined by using the energy dispersive X-ray spectrometer (EDS) (Thermo Fisher Scientific, Waltham, MA, USA). Figure 2a displays the TEM morphology image of the BTBTHP. Figure 2b displays the high-resolution transmission electron microscopy (HRTEM) image of the BTBTHP. In order to better reveal the microstructure of the BTBTHP composite sample, two additional TEM and HRTEM images have been provided, as shown in Figures S9 and S10. Figure 3 shows the EDS elemental scanning map of the BTBTHP. It can be found from Figure 2a that the TEM morphology image of the BTBTHP explicitly described the presence of the BiTmFeSbO_7_ nanoparticle and the BiTmO_3_ nanoparticle which belonged to the BTBTHP. Additionally, the HRTEM stripe image of the interface between the BiTmFeSbO_7_ nanoparticle and the BiTmO_3_ nanoparticle which were marked in Figure 2a can be found from Figure 2b. As a result, the lattice plane spacing d value of 0.305 nm corresponded to the crystal plane index of (222) for the BiTmFeSbO_7_ photocatalyst, and the lattice plane spacing d value of 0.312 nm corresponded to the crystal plane index of (111) for the BiTmO_3_ photocatalyst. The close interfacial contact between the BiTmFeSbO_7_ photocatalyst and the BiTmO_3_ photocatalyst was emphasized, clearly confirming that the coexistence of the BiTmFeSbO_7_ photocatalyst and the BiTmO_3_ photocatalyst was realized in the prepared heterojunction catalyst BTBTHP. The results presented in Figure 3 indicated that the larger particles contained Bi element, Tm element, Fe element, Sb element, and O element. Conversely, the smaller particles did not contain Fe element and Sb element; however, the smaller particles contained Bi element, Tm element, and O element. In accordance with the above analysis, we could draw a conclusion that the larger particles corresponded to the BiTmFeSbO_7_ photocatalyst while the smaller particles corresponded to the BiTmO_3_ photocatalyst. Additionally, Figure 4 displays the EDS spectrum of the BTBTHP. The EDS spectrum which is shown in Figure 4 indicated that the atomic ratio of the Bi element, Tm element, Fe element, Sb element, and O element which were contained in the BTBTHP was approximately 1235:1362:611:613:6179. Based on the above comprehensive results, it could be convincingly concluded that the high-purity BTBTHP was successfully synthesized under the specified preparation parameters.

**Figure 5:**
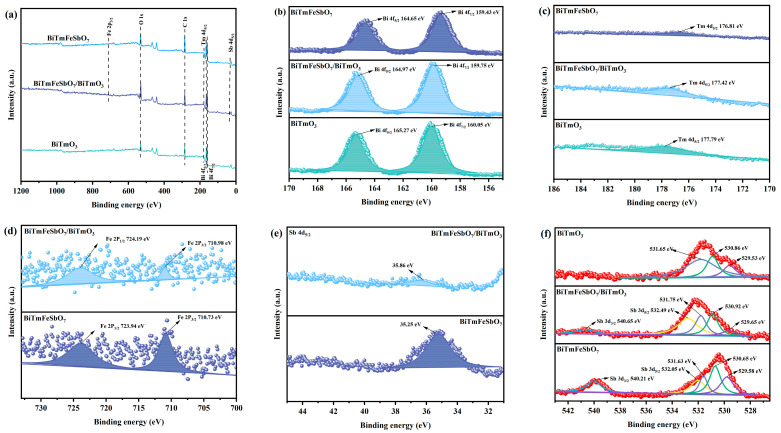
The XPS spectra of the synthesized BiTmFeSbO_7_ photocatalyst, the BiTmO_3_ photocatalyst, and the BTBTHP: (**a**) survey spectrum, (**b**–**f**) high-resolution spectra of Bi 4f, Tm 4d, Fe 2p, Sb 4d, Sb 3d, and O 1S.

The X-ray photoelectron spectrometer (XPS) (VersaProbe, UlVAC-PHI, Chigasaki, Japan) was used for assessing the chemical composition content and oxidation states of the BTBTHP, the BiTmFeSbO_7_ photocatalyst, and the BiTmO_3_ photocatalyst. Figure 5 illustrates the XPS spectra of the BiTmFeSbO_7_ photocatalyst, the BTBTHP, and the BiTmO_3_ photocatalyst. Figure 5a indicates the XPS survey spectra of the BiTmFeSbO_7_ photocatalyst, the BTBTHP, and the BiTmO_3_ photocatalyst. The XPS spectra for the BTBTHP which is presented in Figure 5a revealed the presence of Bi element, Tm element, Fe element, Sb element, and O element, with the carbon peak serving as a calibration reference. Moreover, the XPS spectrum of the BiTmO_3_ photocatalyst was compared with that of the BTBTHP; accordingly, a distinct iron signal and antimony signal were detected in the XPS spectrum of the BTBTHP, indicating that the BTBTHP encompassed the single-phase BiTmFeSbO_7_.

Figure 5b shows the XPS spectral peaks of Bi 4f_7/2_ and Bi 4f_5/2_ which belong to the BiTmFeSbO_7_ photocatalyst, the BiTmO_3_ photocatalyst, and the BTBTHP. As shown in Figure 5b, the Bi 4f_7/2_ peak and the Bi 4f_5/2_ peak of BiTmFeSbO_7_ were positioned at 159.43 eV and 164.65 eV, respectively, while the Bi 4f_7/2_ peak and the Bi 4f_5/2_ peak of the BiTmO_3_ photocatalyst were located at 160.05 eV and 165.27 eV, respectively. The spin–orbit splitting value was 5.22 eV for the above two cases, which was consistent with the +3 oxidation state of the Bi element [59,60,61]. In the BTBTHP, the Bi 4f_7/2_ peak and the Bi 4f_5/2_ peak exhibited a slight shift to 159.75 eV and 164.97 eV.

Figure 5c displays the XPS spectral peak of Tm 4d_5/2_ which belongs to the BiTmFeSbO_7_ photocatalyst, the BiTmO_3_ photocatalyst, and the BTBTHP. In Figure 5c, the 4d_5/2_ peak of Tm derived from the BiTmFeSbO_7_ photocatalyst was located at 176.81 eV and the 4d_5/2_ peak of Tm which originated from the BiTmO_3_ photocatalyst was located at 177.79 eV. Simultaneously, the 4d_5/2_ peak of Tm which originated from the BTBTHP shifted to 177.42 eV. In accordance with Figure 5c, the +3 oxidation state was obtained for the Tm element.

Figure 5d indicates the XPS spectral peaks of Fe 2p_1/2_ and Fe 2p_3/2_ which belong to the BiTmFeSbO_7_ photocatalyst and the BTBTHP. Figure 5d demonstrated that the Fe 2p_1/2_ peak and the Fe 2p_3/2_ peak of the BiTmFeSbO_7_ photocatalyst were located at 723.94 eV and 710.73 eV. As a result, the spin–orbit splitting value which was calculated according to the above 723.94 eV and 710.73 eV was 13.21 eV, indicating that the +3 oxidation state was obtained for the Fe element [59,60,61]. In the BTBTHP, the Fe 2p_1/2_ peak and the Fe 2p_3/2_ peak shifted slightly to 724.19 eV and 710.98 eV, individually.

Figure 5e illustrates the XPS spectral peak of Sb 4d_5/2_ which belongs to the BiTmFeSbO_7_ photocatalyst and the BTBTHP. Figure 5e depicts that the 4d_5/2_ peak of Sb derived from the BiTmFeSbO_7_ photocatalyst was located at 35.25 eV; concurrently, the 4d_5/2_ peak of Sb which came from the BTBTHP underwent a shift to 35.86 eV. According to Figure 5e, the +5 oxidation state was obtained for the Sb element.

Figure 5f exhibits the XPS spectral peaks of Sb 3d_5/2_ and O 1s which belong to the BiTmFeSbO_7_ photocatalyst, the BiTmO_3_ photocatalyst, and the BTBTHP. Figure 5f demonstrated that the Sb 3d_3/2_ peak and the Sb 3d_5/2_ peak of the BiTmFeSbO_7_ photocatalyst were located at 540.21 eV and 532.05 eV, respectively; meanwhile, the Sb 3d_3/2_ peak and the Sb 3d_5/2_ peak of the BTBTHP were positioned at 540.65 eV and 532.75 eV, respectively. It can be found from Figure 5f that the deconvoluted O 1s spectra of the BTBTHP, the BiTmFeSbO_7_ photocatalyst, and the BiTmO_3_ photocatalyst were achieved. In accordance with Figure 5f, the peaks which were observed at 529.65 eV, 529.53 eV, and 529.58 eV were attributable to lattice oxygen [62]. Furthermore, the peaks which were observed at 530.92 eV, 530.86 eV, and 530.65 eV signified the signal which stemmed from adsorbed oxygen [62]. Ultimately, the peaks which were observed at 531.75 eV, 531.65 eV, and 531.63 eV indicated the existence of surface hydroxyl groups [63]. In the light of Figure 5f, the -2 oxidation state was obtained for the oxygen element.

The binding energy of the Bi 4f peak, Tm 4d peak, Fe 2p peak, Sb 4d peak, and Sb 3d peak which existed in the BTBTHP exhibited a positive shift in comparison with that in the BiTmFeSbO_7_ photocatalyst. However, the binding energy of the Bi 4f peak and the Tm 4d peak which existed in the BTBTHP displayed a negative shift compared with that in the BiTmO_3_ photocatalyst, indicating that there was a significant change for the electron density within the heterojunction [64]. Specifically, the above changes indicated that the electron density of Fe and Sb which derived from the BTBTHP decreased; concurrently, the electron density of some Bi and some Tm which came from the BTBTHP increased. Inversely, the electron density of other Bi and other Tm which originated from the BTBTHP decreased [64]. The above phenomenon clearly demonstrates the successful hybridization of the BiTmFeSbO_7_ photocatalyst and the BiTmO_3_ photocatalyst; thereby, the formation of the BTBTHP is validated. The research results further reveal that the intricate electronic interaction mechanism exists inside the BTBTHP. As a result, this interaction is pivotal for enhancing the functional properties of the BTBTHP.

Notably, in accordance with the XPS analysis results, the peak signals which correspond to other secondary phases are not detected in the BTBTHP sample, the BiTmFeSbO_7_ sample, and the BiTmO_3_ sample. Overall, these XPS findings further corroborate the strong chemical interaction between the BiTmFeSbO_7_ photocatalyst and the BiTmO_3_ photocatalyst. These XPS results are consistent with those detection results which are obtained from multiple characterization spectra, including the XRD patterns, the FTIR spectra, the Raman spectra, the TEM morphology image, the HRTEM image, and the EDS element scanning mapping. Eventually, the above detection results collectively verify the compositional characteristic and structural characteristic of the BTBTHP, the BiTmFeSbO_7_ photocatalyst, and the BiTmO_3_ photocatalyst.

#### 3.1.2. Optical Characteristics

**Figure 6:**
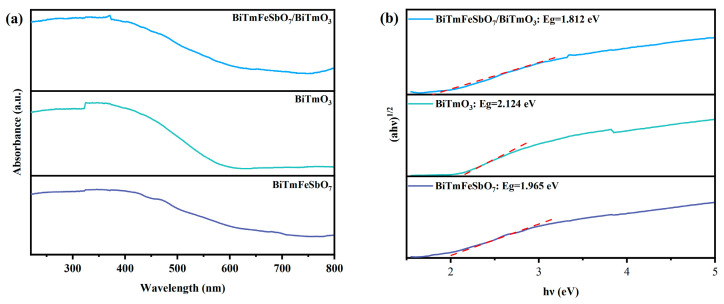
(**a**) The UV–Vis absorption spectra and (**b**) the correlative diagram of (*αhν*)^1/2^ and *hν* of the synthesized BTBTHP, the BiTmFeSbO_7_ photocatalyst, and the BiTmO_3_ photocatalyst.

Figure 6a illustrates the ultraviolet and visible absorption spectra of the BTBTHP, the BiTmO_3_ photocatalyst, and the BiTmFeSbO_7_ photocatalyst. In order to investigate the band structure of the synthesized samples, we conducted an extensive analysis of the diffuse reflectance absorption spectra of the BiTmFeSbO_7_ photocatalyst, the BiTmO_3_ photocatalyst, and the BTBTHP. As depicted in Figure 6a, the absorption edges of the diffuse reflectance spectrum of the BiTmFeSbO_7_ photocatalyst and the BiTmO_3_ photocatalyst were detected at 650 nm and 590 nm, respectively. Notably, the BTBTHP displayed a unique shift absorption edge which is located at approximately 685 nm; as a result, the shift absorption edge wavelength of the BTBTHP presented an obvious red shift characteristic compared with that of the BiTmFeSbO_7_ photocatalyst and the BiTmO_3_ photocatalyst. The aforesaid phenomenon implied that the BTBTHP possessed a stronger light absorption ability than BiTmFeSbO_7_ and BiTmO_3_. Figure 6b shows the correlative diagram of (*αhν*)^1/2^ and *hν* for the synthesized BTBTHP, BiTmFeSbO_7_, and BiTmO_3_. Figure 6b further provided a detailed description for the estimated band gap energy of the BiTmFeSbO_7_ photocatalyst, the BiTmO_3_ photocatalyst, and the BTBTHP. The calculated band gap energy values of the BiTmFeSbO_7_ photocatalyst, the BiTmO_3_ photocatalyst, and the BTBTHP were 1.965 eV, 2.124 eV, and 1.812 eV, respectively. The above band gap energy values are derived from Equation (1) [65]:

(1) (αhν)12 = A(hν−Eg)

Here, *α* represents the absorption coefficient, *ν* represents to the photon energy, *A* denotes the absorbance factor, and *E_g_* is the band gap energy.

**Figure 7:**
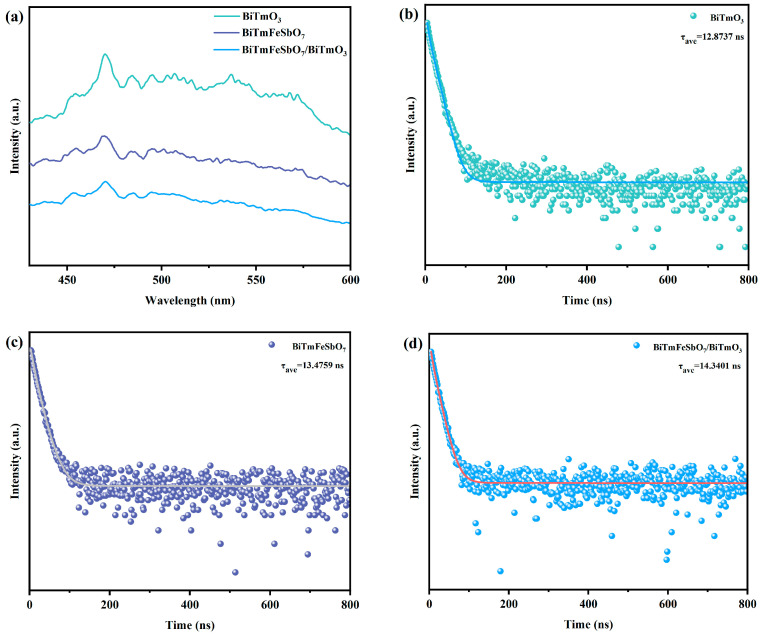
(**a**) The PL spectra of the BTBTHP, the BiTmFeSbO_7_ photocatalyst, and the BiTmO_3_ photocatalyst; (**b**) the TRPL spectrum of the BiTmO_3_ photocatalyst; (**c**) the TRPL spectrum of the BiTmFeSbO_7_ photocatalyst; (**d**) the TRPL spectrum of the BTBTHP.

Figure 7a depicts the photoluminescence (PL) spectrum of the BiTmFeSbO_7_ photocatalyst, the BiTmO_3_ photocatalyst, and the BTBTHP. Figure 7b displays the time-resolved photoluminescence (TRPL) spectrum of the BiTmO_3_ photocatalyst. Figure 7c indicates the TRPL spectrum of the BiTmFeSbO_7_ photocatalyst. Figure 7d shows the TRPL spectrum of the BTBTHP. The photoluminescence spectrum which derived from the BiTmFeSbO_7_ photocatalyst, the BiTmO_3_ photocatalyst, and the BTBTHP served as a means to assess the recombination rate of the PEPH and the photodegradation performance. Meanwhile, the TRPL spectra which originated from the BiTmFeSbO_7_ photocatalyst, the BiTmO_3_ photocatalyst, and the BTBTHP were capable of providing the electron lifetime of the photocatalyst.

Generally, a higher PL intensity implied a faster recombination rate of the PEPH, whereas a lower PL intensity indicated a higher transfer efficiency of the PEPH [66,67]. A relatively high PL intensity was conducive to a decrease in the photocatalytic activity of the BiTmFeSbO_7_ photocatalyst, the BiTmO_3_ photocatalyst, and the BTBTHP. As presented in Figure 7a, BiTmO_3_ exhibited the highest radiation intensity; concurrently, the BiTmFeSbO_7_ photocatalyst possessed an intermediate radiation intensity between the BiTmO_3_ photocatalyst and the BTBTHP; ultimately, the BTBTHP owned the lowest radiation intensity. The above experimental result indicated that the separation rate of the PEPH which derived from the BTBTHP was the fastest, and thereby the photocatalytic removal efficiency for degrading the STZ was enhanced by using the BTBTHP. The above description further demonstrated that the BTBTHP possessed higher photocatalytic activity compared with the BiTmFeSbO_7_ photocatalyst and the BiTmO_3_ photocatalyst. The simulative outcomes of the TRPL spectra which were depicted in Figure 7b (BiTmO_3_), Figure 7c (BiTmFeSbO_7_), and Figure 7d (BTBTHP) were obtained by employing the double exponential decay Equation (2) [68].

(2) $$I(t)=I_{0}+A_{1}\;exp(-\frac{t}{\tau_{1}})+A_{2}\;exp(-\frac{t}{\tau_{2}})$$

In Equation (2) above, *A*_1_ represents the weighting coefficient of the first-order decay times for each decay channel [69], and *A*_2_ represents the weighting coefficient of the second-order decay times for each decay channel [69]. The average lifetime of the PEPH could be computed by using Equation (3) [70].

(3) $$\tau_{ave}=(A_{1}\tau_{1}^{2}+A_{2}\tau_{2}^{2})/(A_{1}\tau_{1}+A_{2}\tau_{2})$$

In accordance with the calculation results, it could be deduced that the lifetime of the BTBTHP (*τ_ave_* = 14.3401 ns) was significantly longer than that of the BiTmFeSbO_7_ photocatalyst (*τ_ave_* = 13.4759 ns) and that of the BiTmO_3_ photocatalyst (*τ_ave_* = 12.8737 ns). These results suggest that the BTBTHP exhibits superior photocatalytic activity in comparison with the BiTmFeSbO_7_ photocatalyst and the BiTmO_3_ photocatalyst.

**Figure 8:**
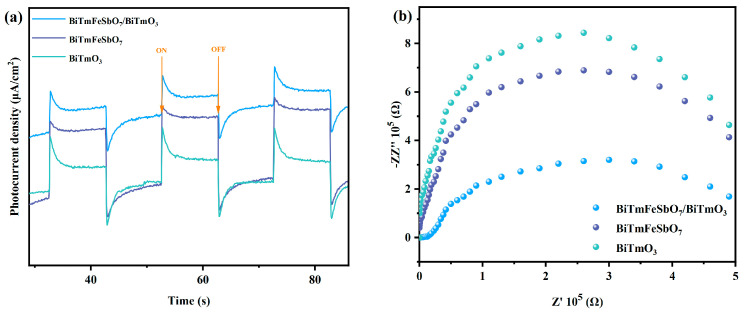
(**a**) The photocurrent density variation curve and (**b**) the EIS plots of the BTBTHP, the BiTmFeSbO_7_ photocatalyst, and the BiTmO_3_ photocatalyst.

Figure 8a presents the photocurrent response spectra of the BiTmFeSbO_7_ sample, the BiTmO_3_ sample, and the BTBTHP sample, obtained from the photocurrent experiments. As is evident from Figure 8a, the BTBTHP exhibited the highest intensity of photocurrent response compared with the BiTmFeSbO_7_ photocatalyst and the BiTmO_3_ photocatalyst. The above phenomenon suggested that the heterojunction structure which was formed by the BiTmFeSbO_7_ photocatalyst and the BiTmO_3_ photocatalyst might facilitate the efficient separation of the PEPH [71,72], and thereby the lifetime of the PEPH was prolonged. Figure 8b displays the Nyquist plots which are obtained from the electrochemical impedance spectroscopy (EIS) measurement experiments. The diameter size value of the semicircle which originates from the mid-frequency region of the Nyquist plot represents the charge transfer resistance (Rct). The Rct reflects the degree of difficulty of the electrochemical reactions which involve electron transfer. A smaller Rct value indicates a greater difficulty for the recombination efficiency of the PEPH and corresponds to a faster electrochemical reaction rate [73,74]. In accordance with the equivalent circuit fitting result, the Rct values of the BTBTHP, the BiTmFeSbO_7_ photocatalyst, and the BiTmO_3_ photocatalyst were determined. The Rct values for the BTBTHP, the BiTmFeSbO_7_ photocatalyst, and the BiTmO_3_ photocatalyst were 4.63 Ω, 4.86 Ω, and 5.32 Ω, respectively. The experimental results indicated that the descending order of the Rct values for the above three catalysts was BTBTHP < the BiTmFeSbO_7_ < the BiTmO; obviously, the BTBTHP exhibited the smallest value of the Rct. The above result demonstrated that the electrochemical reaction rate which was facilitated by using the BTBTHP was higher than that by using the BiTmFeSbO_7_ photocatalyst or the BiTmO_3_ photocatalyst. Thus, the BTBTHP achieved the highest separation efficiency for the PEPH compared with the BiTmFeSbO_7_ photocatalyst and the BiTmO_3_ photocatalyst; as a result, it can be inferred that the BTBTHP possessed higher photocatalytic activity compared with the BiTmFeSbO_7_ photocatalyst and the BiTmO_3_ photocatalyst. These photocurrent experimental outcomes are consistent with previous PL experimental results and previous TRPL experimental results, further demonstrating that the BTBTHP possesses superior photocatalytic activity.

### 3.2. Examination of Photocatalytic Degradation Efficiency

#### 3.2.1. Photocatalytic Degradation of the STZ

**Figure 9:**
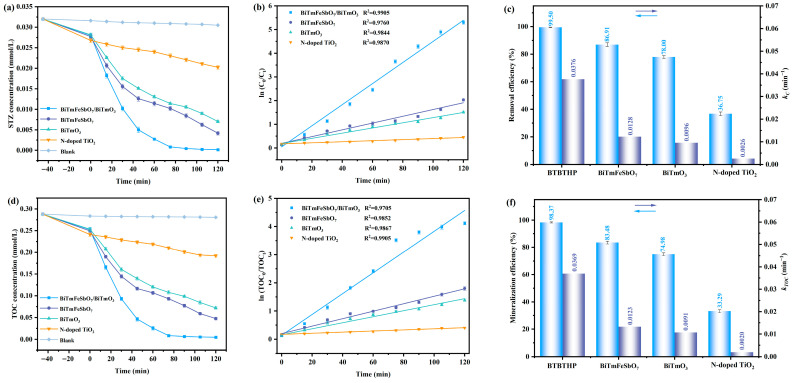
(**a**) The curves of the STZ concentration versus VLIRN time for the photocatalytic degradation of the STZ by using the BTBTHP, the BiTmFeSbO_7_ photocatalyst, the BiTmO_3_ photocatalyst, and the N-T photocatalyst; (**b**) The simulative results of ln(C_0_/C_t_) versus VLIRN time by using the BTBTHP, the BiTmFeSbO_7_ photocatalyst, the BiTmO_3_ photocatalyst, and the N-T photocatalyst; (**c**) The photodegradation rate of the STZ and the kinetic constants after VLIRN of 120 min by using the BTBTHP, the BiTmFeSbO_7_ photocatalyst, the BiTmO_3_ photocatalyst, and the N-T photocatalyst; (**d**) The curves of the TOC concentration versus VLIRN time for the photocatalytic degradation of the STZ by using the BTBTHP, the BiTmFeSbO_7_ photocatalyst, the BiTmO_3_ photocatalyst, and the N-T photocatalyst; (**e**) The simulative results of the ln(TOC_0_/TOC_t_) versus VLIRN time during the degradation process of the STZ by using the BTBTHP, the BiTmFeSbO_7_ photocatalyst, the BiTmO_3_ photocatalyst, and the N-T photocatalyst; (**f**) The mineralization efficiency of the TOC concentration and the kinetic constants after VLIRN of 120 min by using the BTBTHP, the BiTmFeSbO_7_ photocatalyst, the BiTmO_3_ photocatalyst, and the N-T photocatalyst.

The photocatalytic performance of the STZ under VLIRN by using the BTBTHP, the BiTmFeSbO_7_ photocatalyst, the BiTmO_3_ photocatalyst, and the N-T photocatalyst is depicted in Figure 9. Figure 9a shows the variation curve which is caused by the effect of the STZ concentration on the VLIRN time for the photocatalytic degradation of the STZ by using the BTBTHP, the BiTmFeSbO_7_ photocatalyst, the BiTmO_3_ photocatalyst, or the N-T photocatalyst under the condition of VLIRN. The solution was initially stirred in darkness for 45 min to reach adsorption–desorption equilibrium. It can be seen from Figure 9a that the concentration of the STZ gradually decreased with the extension of VLIRN time by using the BTBTHP, the BiTmFeSbO_7_ photocatalyst, the BiTmO_3_ photocatalyst, or the N-T photocatalyst. The BTBTHP possessed the highest degradation efficiency for the STZ compared with the BiTmFeSbO_7_ photocatalyst, the BiTmO_3_ photocatalyst, or the N-T photocatalyst.

Figure 9b shows the mimetic variation curve which is formed according to the effect of the ln (C_0_/C_t_) on the VLIRN time during the photocatalytic degradation process of the STZ by using the BTBTHP, the BiTmFeSbO_7_ photocatalyst, the BiTmO_3_ photocatalyst, and the N-T photocatalyst. As shown in Figure 9b, the mimetic variation curve caused by the BTBTHP, the BiTmFeSbO_7_ photocatalyst, the BiTmO_3_ photocatalyst, and the N-T photocatalyst conformed to the first-order kinetic model. The kinetic constant *K_C_* was calculated using the equation *ln*(*C*_0_/*C_t_*) = *K_C_t*, where *C*_0_ was the initial concentration of the STZ; concurrently, *C_t_* represented the concentration of the STZ at a specific VLIRN time; moreover, *t* meant the VLIRN time.

Figure 9c summarizes the photodegradation efficiency of the STZ and the kinetic constant *K_C_* after VLIRN of 120 min by using the BTBTHP, the BiTmFeSbO_7_ photocatalyst, the BiTmO_3_ photocatalyst, and the N-T photocatalyst. Compared with the BiTmFeSbO_7_ photocatalyst, the BiTmO_3_ photocatalyst, and the N-T photocatalyst, BTBTHP had the highest photodegradation efficiency for the STZ. The removal rates of the STZ using the BTBTHP, the BiTmFeSbO_7_ photocatalyst, the BiTmO_3_ photocatalyst, and the N-T photocatalyst were 99.50%, 86.91%, 78.00%, and 36.75%, respectively, under the condition of VLIRN. The removal rate of the STZ by using the BTBTHP was 1.14 times that of using the BiTmFeSbO_7_ photocatalyst, and 1.28 times that of using the BiTmO_3_ photocatalyst, and 2.71 times that of using the N-T photocatalyst. Furthermore, the reaction rate for degrading the STZ by using the BTBTHP, the BiTmFeSbO_7_ photocatalyst, the BiTmO_3_ photocatalyst, or the N-T photocatalyst was 3.87 × 10^−9^ mol·L^−1^·s^−1^, 3.27 × 10^−9^ mol·L^−1^·s^−1^, 2.93 × 10^−9^ mol·L^−1^·s^−1^, or 9.09 × 10^−10^ mol·L^−1^·s^−1^, respectively. The incident photon flux which was obtained after VLIRN was measured to be 4.76 × 10^−6^ Einstein L^−1^·s^−1^ by using a radiometer. In accordance with Equation (4), the photon efficiency (PHE) value for degrading the STZ by using the BTBTHP, the BiTmFeSbO_7_ photocatalyst, the BiTmO_3_ photocatalyst, or the N-T photocatalyst was calculated to be 0.0813%, 0.0687%, 0.0615%, or 0.0191%, respectively. The first-order kinetic constant *K_C_* was calculated to be 0.0376 min^−1^, 0.0128 min^−1^, 0.0096 min^−1^, or 0.0026 min^−1^ during the degradation process of the STZ by using the BTBTHP, the BiTmFeSbO_7_ photocatalyst, the BiTmO_3_ photocatalyst, or the N-T photocatalyst, respectively. Obviously, the value of the kinetic constant *K_C_* during the degradation process of the STZ by using the BTBTHP was the highest compared with those of using the BiTmFeSbO_7_ photocatalyst, the BiTmO_3_ photocatalyst, or the N-T photocatalyst.

The PHE was calculated according to Equation (4):

(4) $$\phi =R/I_{0}$$

As to Equation (4), ϕ represented the photon efficiency; concurrently, *R* was the degradation rate of the STZ; moreover, $I_{0}$ was the photon flux.

Figure 9d shows the variation curve which is formed in accordance with the effect of the total organic carbon (TOC) concentration on the VLIRN time during the photocatalytic degradation process of the STZ by using the BTBTHP, the BiTmFeSbO_7_ photocatalyst, the BiTmO_3_ photocatalyst, and the N-T photocatalyst. The results in Figure 9d were consistent with the degradation results of the STZ. As shown in Figure 9d, the TOC concentration gradually decreased with increasing the VLIRN time; obviously, the BTBTHP exhibited the highest mineralization efficiency for removing the TOC concentration during the photocatalytic degradation process of the STZ compared with the BiTmFeSbO_7_ photocatalyst, the BiTmO_3_ photocatalyst, and the N-T photocatalyst.

Figure 9e shows the simulative results of the variation curve which is formed according to the effect of the ln(TOC_0_/TOC_t_) on the VLIRN time during the photocatalytic degradation process of the STZ by using the BTBTHP, the BiTmFeSbO_7_ photocatalyst, the BiTmO_3_ photocatalyst, and the N-T photocatalyst. It can be found from Figure 9e that the simulative results conformed to the first-order kinetic model. The kinetic constants were calculated by using the equation of *ln*(*TOC*_0_/*TOC_t_*) = *K_TOC_t*, where *TOC*_0_ was the initial concentration of TOC; simultaneously, *TOC* was the reaction concentration of TOC at a specific VLIRN time; in addition, *t* represented the VLIRN time.

Figure 9f summarizes the mineralization efficiency of TOC concentration and the kinetic constant *K_TOC_* during the photocatalytic degradation process of the STZ after VLIRN of 120 min by using the BTBTHP, the BiTmFeSbO_7_ photocatalyst, the BiTmO_3_ photocatalyst, and the N-T photocatalyst. The BTBTHP possessed the highest mineralization rate for removing the TOC concentration compared with the BiTmFeSbO_7_ photocatalyst, the BiTmO_3_ photocatalyst, and the N-T photocatalyst. In accordance with Figure 9f, the removal rates of the TOC concentration by using the BTBTHP, the BiTmFeSbO_7_ photocatalyst, the BiTmO_3_ photocatalyst, and the N-T photocatalyst were 98.37%, 83.48%, 74.98%, and 33.29% after VLIRN of 120 min. The mineralization rate for removing the TOC concentration by using the BTBTHP was 1.18 times that of using the BiTmFeSbO_7_ photocatalyst, 1.31 times that of using the BiTmO_3_ photocatalyst, and 2.95 times that of using the N-T photocatalyst. The first-order kinetic constants *K_TOC_* were 0.0369 min^−1^, 0.0123 min^−1^, 0.0091 min^−1^, and 0.0020 min^−1^ when the BTBTHP, the BiTmFeSbO_7_ photocatalyst, the BiTmO_3_ photocatalyst, and the N-T photocatalyst were utilized for degrading the STZ. Obviously, the value of the kinetic constant *K_TOC_* during the photocatalytic degradation process of the STZ by using the BTBTHP was the highest compared with those of using the BiTmFeSbO_7_ photocatalyst, the BiTmO_3_ photocatalyst, and the N-T photocatalyst.

**Figure 10:**
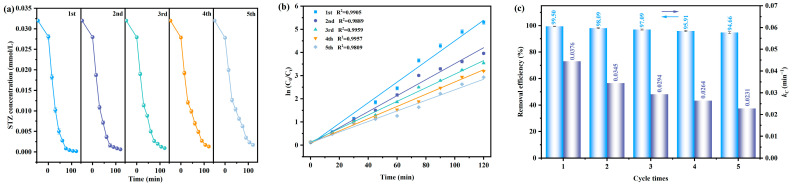

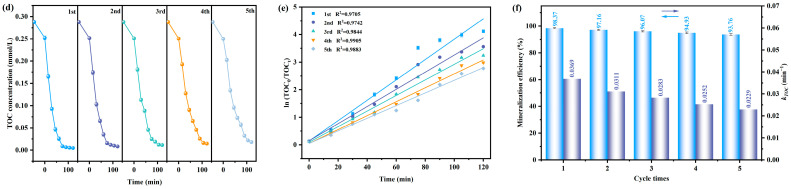
(**a**) The effect of the STZ concentration on the VLIRN time during quintic cyclic experiments by using the BTBTHP; (**b**) The effect of the ln(C_0_/C_t_) on VLIRN time during the quintic cyclic degradation process of the STZ by using the BTBTHP; (**c**) The removal efficiencies of the STZ and the kinetic constants for degrading the STZ during quintic cyclic experiments by using the BTBTHP under the condition of VLIRN; (**d**) The effect of the TOC concentration on the VLIRN time during quintic cyclic experiments by using the BTBTHP; (**e**) The effect of the ln(TOC_0_/TOC_t_) on VLIRN time during the quintic cyclic degradation process of the STZ by using the BTBTHP; (**f**) The mineralization efficiency of the TOC concentration and the kinetic constants deriving from the contribution of ln(TOC_0_/TOC_t_) and VLIRN time during quintic cyclic experiments by using the BTBTHP.

In order to evaluate the practical durability and reusability of the synthesized photocatalysts, the cyclic performance tests are conducted by using the synthesized BTBTHP and the results are shown in Figure 10a–f. Figure 10a shows the effect of the STZ concentration on the VLIRN time during quintic cyclic experiments by using the BTBTHP. Figure 10b shows the effect of the ln(C_0_/C_t_) on VLIRN time during the quintic cyclic degradation process of the STZ by using the BTBTHP. Figure 10c shows the removal efficiencies of the STZ and the kinetic constants for degrading the STZ during quintic cyclic experiments by using the BTBTHP under the condition of VLIRN. Figure 10d shows the effect of the TOC concentration on the VLIRN time during quintic cyclic experiments by using the BTBTHP. Figure 10e shows the effect of the ln(TOC_0_/TOC_t_) on VLIRN time during the quintic cyclic degradation process of the STZ by using the BTBTHP. Figure 10f shows the mineralization efficiency of the TOC concentration and the kinetic constants deriving from the contribution of ln(TOC_0_/TOC_t_) and VLIRN time during quintic cyclic experiments by using the BTBTHP. Notably, the BTBTHP exhibited excellent stability during five successive photocatalytic cyclic degradation experiments. Specifically, the degradation efficiency of the STZ by using the BTBTHP after quintic cyclic experiments under the condition of VLIRN was above 94%; concurrently, the mineralization efficiency of the TOC concentration by using the BTBTHP after quintic cyclic experiments under the condition of VLIRN was above 93%. These findings indicated that the BTBTHP possessed significant application potential in the domain of wastewater treatment for removing the STZ pollutant.

In order to evaluate the structural stability of the BTBTHP, the BTBTHP which was recovered after five cyclical experiments for degrading the STZ was characterized by using the X-ray diffractometer, the ultraviolet and visible spectrophotometer, and the scanning electron microscope. The corresponding results are presented in Figures S11–S14. Figure S11 shows the XRD spectrum of the BTBTHP after the quintic cyclic degradation process of the STZ by using the BTBTHP under the condition of VLIRN. Figure S12 displays the ultraviolet and visible absorption spectrum of the BTBTHP after the quintic cyclic degradation process of the STZ by using the BTBTHP under the condition of VLIRN. Figure S13 indicates the SEM morphology pattern of the BTBTHP after the quintic cyclic degradation process of the STZ by using the BTBTHP under the condition of VLIRN. Figure S14 exhibits the EDS element scanning mapping of the BTBTHP after the quintic cyclic degradation process of the STZ by using the BTBTHP under the condition of VLIRN (Bi, Tm, Fe, Sb, and O from BiTmFeSbO_7_; simultaneously, Bi, Tm, and O from BiTmO_3_). As shown in Figure S11, the structure of the BTBTHP remained stable after five consecutive cyclical experiments for degrading the STZ. It can be found from Figure S12 that the ultraviolet and visible absorption spectrum of the recovered BTBTHP did not exhibit significant changes; concurrently, the intrinsic transition absorption edge of the BTBTHP remained unaltered and still situated within the visible light waveband region. Figure S13 indicated that the particle structure of the BTBTHP did not collapse and maintained a perfect crystal texture; meanwhile, the granulometric configuration which derived from the BTBTHP was not changed. Moreover, as shown in Figure S14, the composition content of the BTBTHP was not varied; simultaneously, the metallic simple substance did not precipitate. The molar ratio of the elements which were contained within the recovered BTBTHP after five consecutive cyclical experiments for degrading the STZ was determined to be Bi:Tm:Fe:Sb:O = 1229:1357:615:621:6158. In conclusion, these results demonstrate that the structure of the BTBTHP remains stable after five consecutive cyclical experiments for degrading the STZ, indicating that the BTBTHP possessed excellent recyclability.

When the initial concentration of the STZ was 0.032 mmol/L, this study investigated the influence of different BTBTHP dosages on the removal efficiency of the STZ during the photocatalytic degradation process of the STZ under the condition of VLIRN. Figure S15 demonstrates the effect of different BTBTHP dosages on the degradation efficiency of the STZ after VLIRN of 120 min. It can be found from Figure S15 that the degradation removal rate of the STZ reached a maximal value of 99.5% when the concentration of the BTBTHP was 0.500 g/L; however, when the concentration of the BTBTHP was further increased, the degradation efficiency of the STZ progressively decreased. The above phenomenon may be attributed to the fact that higher concentrations of the BTBTHP can shield the incident visible light; correspondingly, the accessible surface area for the interactions which occur among visible light, the STZ, and the BTBTHP is reduced; consequently, the utilization efficiency of the active sites which exist on the catalyst surface is decreased.

The initial solution’s pH value typically influences the photocatalytic activity of the BTBTHP. Therefore, this study examined the degradation efficiency of the STZ by using the BTBTHP during the photocatalytic degradation process of the STZ under the condition of VLIRN at the initial pH values of 3, 5, 7, 9, or 11. Figure S16 presents the effect of different pH values on the degradation efficiency of the STZ by using the BTBTHP during the photocatalytic degradation process of the STZ under the condition of VLIRN. As shown in Figure S16, the systematic trend for the photocatalytic activity of the BTBTHP was analyzed. The removal rates of the STZ at pH values of 3, 5, 7, 9, and 11 were 91.31%, 93.34%, 99.50%, 96.34%, and 76.42% by using the BTBTHP after VLIRN of 120 min. These results demonstrate that neutral and weakly alkaline conditions are conducive to enhancing the removal rate of the STZ; conversely, the removal rate of the STZ decreased under acidic conditions. Based on the above results, a neutral pH value of 7 was selected as the experimental condition for this study.

In order to investigate the effect of the anion category on the removal rate for the STZ by using the BTBTHP under the condition of VLIRN, ultrapure water which contained four different anions was introduced into the photocatalytic reaction system; meanwhile, every anion possessed a concentration of 10 mmol/L. Figure S17 illustrates the influence of different anions on the degradation efficiency of the STZ during the photocatalytic degradation process of the STZ by using the BTBTHP under the condition of VLIRN. As shown in Figure S17, after the addition of SO_4_^2−^, NO_3_^−^, Cl^−^ or CO_3_^2−^, the degradation efficiency of the STZ declined by using the BTBTHP under the condition of VLIRN. After VLIRN of 120 min, the removal rate of the STZ reached 99.5% during the photocatalytic degradation process of the STZ by using the BTBTHP. Under the identical conditions, the introduction of 10 mmol/L SO_4_^2−^ into the photocatalytic reaction system resulted in a removal rate of 98.7% for the STZ by using the BTBTHP; moreover, the removal rate of the STZ was 96.9% with the addition of 10 mmol/L NO_3_^−^ by using the BTBTHP under the condition of VLIRN, concurrently, the photodegradation removal rate of the STZ dropped to 60.3% with the augmentation of 10 mmol/L Cl^−^, eventually, the degradation removal rate of the STZ was 65.6% with the increment of 10 mmol/L CO_3_^2−^. The aforementioned results indicate that the Cl^−^ ions which are contained within water sources have a significant impact on the degradation efficiency of the STZ. Higher concentrations of the chloride ion lead to greater consumption of the hydroxyl radicals and the photogenerated holes, ultimately, a reduction in the active radicals is realized in the wastewater system, thereby, the photocatalytic reaction is hindered, finally, a decrease for the removal rate of the STZ is realized. In contrast, water sources which contained high concentrations of SO_4_^2−^ and NO_3_^−^ exhibited a weaker inhibitory effect during the photocatalytic degradation process of the STZ. This is primarily because SO_4_^2−^ or NO_3_^−^ only consume the photogenerated holes without affecting the generation of the hydroxyl radicals, thus a lesser inhibition degree for the overall photocatalytic degradation of the STZ appeared.

This study investigated the influence of the initial STZ concentration on the photocatalytic degradation efficiency of the STZ by using the BTBTHP under the condition of VLIRN. Figure S18 demonstrates the effect of different initial STZ concentrations on the degradation efficiency of the STZ by using the BTBTHP under the condition of VLIRN. As shown in Figure S18, when the initial concentrations of the STZ were 0.008 mmol/L, 0.016 mmol/L, 0.032 mmol/L and 0.064 mmol/L, the corresponding degradation efficiencies of the STZ reached 99.63%, 99.57%, 99.50% and 89.65%, respectively. These results indicate that the high initial concentrations of the STZ lead to rapid saturation of the active sites which existed on the BTBTHP surface; meanwhile, the substantial quantity of the generated intermediate products further occupies these active sites, consequently, the continuous generation of the active radicals was hindered. When the BTBTHP was employed under the condition of VLIRN, the degradation reactions proceeded too rapidly for accurate experimental observation at initial STZ concentration of 0.008 mmol/L or 0.016 mmol/L. Therefore, an initial STZ concentration of 0.032 mmol/L was selected as the standard condition for the photocatalytic experiments in this study.

**Figure 11:**
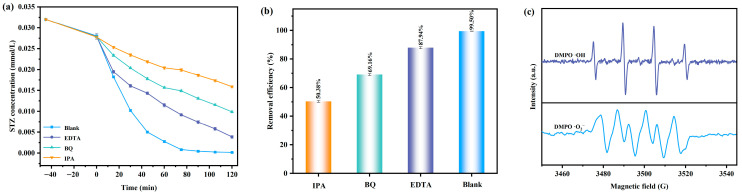
(**a**) The change curves deriving from the effect of the STZ concentration on the VLIRN time with the addition of the radical scavenger such as IPA, BQ, and EDTA; (**b**) The removal efficiency of the STZ during the degradation process of the STZ by using the BTBTHP with IPA, BQ, and EDTA as scavengers under the condition of VLIRN; (**c**) The EPR spectra of DMPO•O_2_^-^ and DMPO•OH during the degradation process of the STZ by using the BTBTHP under the condition of VLIRN.

In order to investigate the active substances which were generated during the photodegradation process of the STZ by using the BTBTHP under the condition of VLIRN, the experiments for trapping the radicals were conducted. The hydroxyl radicals (•OH) were captured by using the IPA; concurrently, the superoxide anions (•O_2_^−^) were captured by using the BQ; moreover, the photoinduced holes (h^+^) were captured by using the EDTA. Figure 11a shows the change curves which derive from the effect of the STZ concentration on the VLIRN time with the addition of the radical scavengers such as IPA, BQ, and EDTA. Figure 11b shows the removal efficiency of the STZ during the photocatalytic degradation process of the STZ by using the BTBTHP with IPA, BQ, and EDTA as scavengers under the condition of VLIRN. For the sake of eliminating the •OH, the •O_2_^−^, and the h^+^ during the photocatalytic degradation process of the STZ, the radical scavengers such as IPA, BQ, and EDTA were introduced at the initial stage of the photodegradation process of the STZ. The results which are displayed in Figure 11a,b indicated that the removal efficiency of the STZ was 99.50% by using the BTBTHP under the condition of VLIRN without using the scavenger; simultaneously, the removal rate of the STZ decreased to 50.38% in the presence of the IPA by using the BTBTHP under the condition of VLIRN; moreover, the removal rate of the STZ decreased to 69.16% in the presence of the BQ by using the BTBTHP under the condition of VLIRN; ultimately, the removal rate of the STZ decreased to 87.94% in the presence of the EDTA by using the BTBTHP under the condition of VLIRN. The above results indicated that •O_2_^−^, •OH, and h^+^ could be utilized as reactive radicals for involvement in the photocatalytic degradation of the STZ. The above experimental results demonstrated that the hydroxyl radicals exhibited the strongest oxidative removal capacity for degrading the STZ compared with the superoxide anions and the photoinduced holes during the photocatalytic degradation process of the STZ by using the BTBTHP under the condition of VLIRN. Among the three reactive radicals, the oxidative removal capacity for degrading the STZ decreased in the descending order of •OH > •O_2_^−^ > h^+^. Figure 11c shows the electron paramagnetic resonance (EPR) spectrum of the DMPO•O_2_^-^ and the DMPO•OH during the photocatalytic degradation process of the STZ by using the BTBTHP under the condition of VLIRN. As depicted in Figure 11c, after VLIRN for 10 min, the EPR spectrum exhibited a distinct DMPO•OH signal which presented a four lines signal with an intensity ratio of 1:2:2:1 which confirmed the presence of the hydroxyl radicals [75]. Additionally, the EPR spectrum displayed a distinct DMPO•O_2_^−^ signal which followed four prominent peaks with an intensity ratio of 1:1:1:1 [75]. The aforementioned experimental results demonstrated that the hydroxyl radicals and the superoxide anions were simultaneously generated during the photodegradation process of the STZ by using the BTBTHP under the condition of VLIRN. The yield of the reactive radicals was determined by the relative intensity of the EPR signals; therefore, it can be concluded that the yield of the hydroxyl radicals was significantly higher than the yield of the superoxide anions [75,76]. The aforementioned experimental results were consistent with the results which originated from the previous radical scavenger experiments; ultimately, it was corroborated that the involvement of the hydroxyl radicals and the superoxide anions during the photocatalytic degradation process of the STZ was realized.

#### 3.2.2. Comparison of the Photocatalytic Activity

In order to demonstrate the innovative value and application potential of this research, a comprehensive comparative analysis which corresponded to the removal rate of the STZ by using different photocatalysts was conducted. Table 1 displays the comparison results for the photocatalytic activity of the BTBTHP with other reported photocatalysts during the photodegradation process of the STZ under the condition of VLIRN. As systematically summarized in Table 1, the results clearly indicated that the photocatalytic activity of the BTBTHP outperformed that of other photocatalysts significantly. The exceptional effectiveness of the BTBTHP for the photocatalytic degradation of the STZ has been demonstrated under the condition of VLIRN, indicating that the BTBTHP highlighted the superior activity compared with other existing photocatalysts.

#### 3.2.3. Possible Photocatalytic Mechanism of the STZ

**Figure 12:**
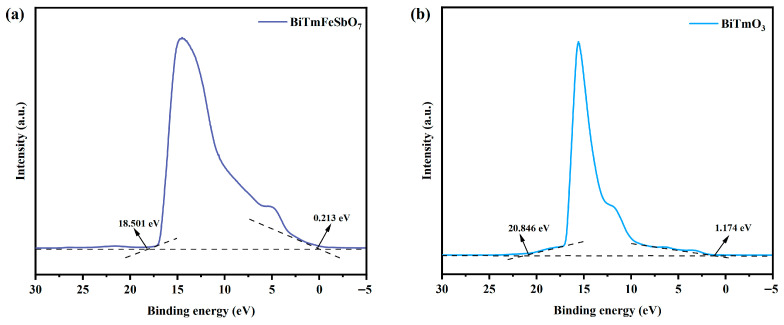
The UPS spectra of (**a**) the BiTmFeSbO_7_ photocatalyst and (**b**) the BiTmO_3_ photocatalyst.

Figure 12 presents the ultraviolet photoelectron spectrum (UPS) of BiTmFeSbO_7_ and BiTmO_3_. The UPS analysis was conducted for determining the ionization potential of BiTmFeSbO_7_ or BiTmO_3_ accurately. As depicted in Figure 12, the onset binding energy (Ei) and the cutoff binding energy (Ecutoff) of BiTmFeSbO_7_ were 0.213 eV and 18.501 eV, respectively; simultaneously, the Ei and the Ecutoff of BiTmO_3_ were 1.174 eV and 20.846 eV, respectively [82]. According to the excitation energy of 21.2 eV, the valence band (VB) ionization potentials of the BiTmO_3_ and the BiTmFeSbO_7_ were ascertained to be 1.528 eV and 2.912 eV, respectively [83,84]. Subsequently, the conduction band (CB) potentials of BiTmO_3_ and BiTmFeSbO_7_ were determined to be −0.596 eV and 0.947 eV, respectively.

**Figure 13:**
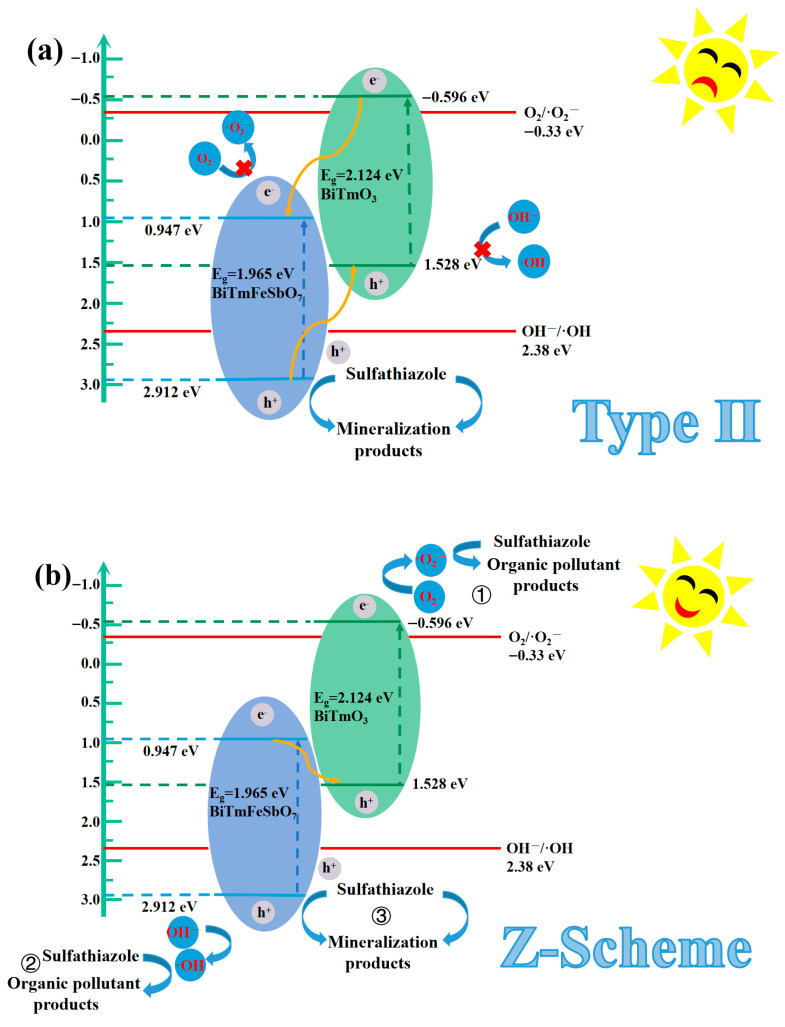
Plausible photodegradation mechanism of the STZ by using the BTBTHP under the condition of VLIRN: (**a**) conventional type II; (**b**) direct Z-scheme.

After determining the VB ionization potentials of the BiTmFeSbO_7_ and the BiTmO_3_, the CB potentials of the BiTmFeSbO_7_ and the BiTmO_3_ could be obtained. Concurrently, an in-depth investigation into the photocatalytic mechanism for degrading the STZ by using the BTBTHP was carried out. Consequently, two possible heterojunction photocatalysts were proposed: the conventional type-II heterojunction photocatalyst and the direct Z-scheme heterojunction photocatalyst [85]. Figure 13 exhibits the plausible photodegradation mechanism of the STZ by using the BTBTHP under the condition of VLIRN. As depicted in Figure 13a, in the structure of the conventional type-II heterojunction photocatalyst BiTmFeSbO_7/_BiTmO_3_, owing to the potential difference in the heterojunction photocatalyst BiTmFeSbO_7_/BiTmO_3_, the photogenerated electrons which existed in the CB position of the BiTmO_3_ would transfer to the CB position of the BiTmFeSbO_7_; as a result, the photogenerated electrons would accumulate in the CB position of the BiTmFeSbO_7_; thereby, the reduction potential for the CB position of the BiTmFeSbO_7_ decreased. Concurrently, the photogenerated holes which resided in the VB position of the BiTmFeSbO_7_ would migrate to the VB position of the BiTmO_3_. As a result, the photogenerated holes accumulated in the VB position of the BiTmO_3_; correspondingly, the oxidation potential for the VB position of the BiTmO_3_ reduced. In accordance with the above assumption, the CB potential 0.947 eV for BiTmFeSbO_7_ was more positive than the O_2_/•O_2_^−^ potential of −0.33 eV (vs. NHE), and thus the photogenerated electrons which existed in the CB position of the BiTmFeSbO_7_ could not effectively react with O_2_ for generating •O_2_^−^ [86]. Similarly, the negative VB potential value of 1.528 eV for the BiTmO_3_ was lower than the potential value of 2.38 eV (vs. NHE) which corresponded to (OH^−^/•OH); as a result, the above result would inhibit the generation of •OH in the VB position of the BiTmO_3_ [87]. Nevertheless, these findings conflicted with the experimental results of •O_2_^−^ radicals and •OH radicals which were obtained from the trapping radicals experiments and the EPR experiments.

Based on the foregoing results, we hypothesized that the direct Z-scheme heterojunction provided a reasonable explanation for the photocatalytic degradation mechanism of the STZ with the BiTmFeSbO_7_/BiTmO_3_ heterojunction as the photocatalyst under the condition of VLIRN in this study. Figure 13b presented the Z-scheme heterojunction mechanism for degrading the STZ by using the BTBTHP under the condition of VLIRN. As a result, the photogenerated electrons migrated from the CB position which had a potential of 0.947 eV for BiTmFeSbO_7_ to the VB which possessed a potential of 1.528 eV for BiTmO_3_. The above migration process effectively facilitated the separation of the photogenerated electrons and the photogenerated holes; concurrently, high oxidation potential and high reduction potential were maintained; thereby, the high oxidation potential and the high reduction potential were conducive to improving the photocatalytic degradation efficiency of the STZ. Consequently, the structure of the BTBTHP enabled the photoinduced electrons which existed in the CB position of the BiTmO_3_ to interact with O_2_ for generating the •O_2_^−^; thereby, the target pollutant STZ was degraded by using the •O_2_^−^ (① in Figure 13b). Simultaneously, the h^+^ which existed in the VB position of the BiTmFeSbO_7_ reacted with OH^−^ for producing the •OH which played a pivotal role during the photocatalytic degradation process of the pollutant STZ (② in Figure 13b). Furthermore, the photogenerated holes (h^+^) which existed in the VB position of both BiTmFeSbO_7_ and BiTmO_3_ could directly catalyze and oxidize the STZ due to the inherent potent oxidation capacity of the photoinduced holes; subsequently, the photoinduced holes participated in the subsequent degradation process of the STZ (③ in Figure 13b). It was noteworthy that the above analysis results were consistent with the findings which derived from the radical trapping experiments and the EPR experiments, as depicted in Figure 11.

Consequently, the above analysis results reveal that the charge transfer pathway which belongs to the heterojunction which is composed of BiTmFeSbO_7_ and BiTmO_3_ follows the direct Z-scheme mechanism under the condition of VLIRN; as a result, the photocatalytic activity of the BTBTHP is enhanced.

#### 3.2.4. Possible Contribution of Each Element for the Photocatalytic Degradation of the STZ

The distortion of lone pair electrons which exist in the bismuth 6s orbital can induce hybridization between the Bi 6s orbital and the O 1s orbital in the valence band of the photocatalyst. This orbital interaction contributes to a decrescence of the band gap width and facilitates the migration rate of the PEPH; therefore, the visible light response capability of the BTBTHP is enhanced [88,89]. Furthermore, the incorporation of the thulium (Tm) element plays a similar role which originates from other rare-earth dopants. The f-f electronic transition within the 4f orbit of the Tm element effectively broadens the light absorption range; thereby, more efficient utilization of the solar light is realized and, simultaneously, the separation efficiency of the PEPH is enhanced [90,91,92]. The drawing in iron (Fe) element serves as the electron traps and the active sites; therefore, substantial absorption of visible light energy is realized through localized interactions within the photocatalyst. Fe^3+^ ions will function as the trapping centers which can capture and transfer the PEPH; as a result, the recombination probability of the PEPH may be effectively suppressed [93,94,95]. Meanwhile, the antimony (Sb) element possesses ns^2^ electron lone pairs, which significantly enhance charge carrier concentration and promote the formation of the oxygen vacancies; consequently, the photoelectronic properties of the BTBTHP can be improved. The doping of the antimony element achieves a contraction of the optical band gap; as a result, the recombination efficiency of the PEPH may be effectively suppressed [96,97,98]. Consequently, the above results promote the generation of the hydroxyl radicals and accelerate the overall photocatalytic reaction rate. The formation of a Z-scheme heterojunction which derives from the BiTmFeSbO_7_ photocatalyst and the BiTmO_3_ photocatalyst constitutes the key factor for achieving synergistic enhancement. This Z-scheme heterojunction BiTmFeSbO_7_/BiTmO_3_ enables the PEPH to separate effectively; therefore, a great quantity of active species which possess high oxidizing ability is generated during the photocatalytic degradation process of the STZ under the condition of VLIRN; correspondingly, the above active species contains the hydroxyl radicals and the superoxide anions. Consequently, the above mechanism leads to the highly efficient degradation removal rate of the STZ by using the BTBTHP under the condition of VLIRN.

The removal rate of the STZ by using Sb-doped N-T was 48.36% after VLIRN of 120 min. The removal rate of the STZ by using Sb-doped N-T was higher compared with those of using Bi-doped N-T, Tm-doped N-T, and Fe-doped N-T. Therefore, Sb possessed the most superior properties for degrading the STZ compared with Fe, Bi, and Tm under the condition of VLIRN.

#### 3.2.5. Possible Degradation Pathways of the STZ

**Figure 14:**
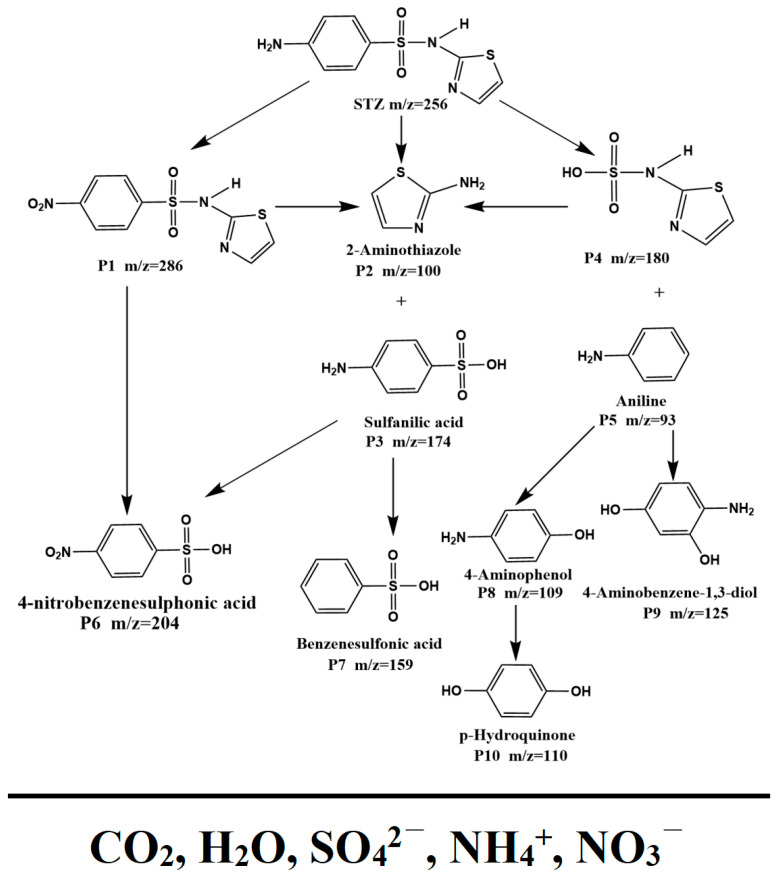
The possible photodegradation pathways for the STZ under the condition of VLIRN by using the BTBTHP.

Figure 14 displays the possible photodegradation pathways for the STZ under the condition of VLIRN by using the BTBTHP. Based on the experimental results which were accomplished by using the liquid chromatograph–mass spectrometer (LC–MS) and the existing literature, three different potential degradation pathways of the STZ (*m*/*z* = 256) were proposed, as shown in Figure 14 [99,100,101,102]. In Pathway 1, the amino group which existed on the benzene ring of the STZ was oxidized to a nitro group; as a result, product P1 was generated with *m*/*z* = 286. Subsequently, the S-N bond was broken under the attack of the active radicals; thereby, 4-nitrobenzenesulfonic acid P6 (*m*/*z* = 204) and 2-aminothiazole P2 (*m*/*z* = 100) were generated [99]. In Pathway 2, the S-N bond was broken under the action of the active radicals; as a result, 2-aminothiazole P2 (*m*/*z* = 100) and sulfanilic acid P3 (*m*/*z* = 174) were produced. The sulfanilic acid P3 (*m*/*z* = 174) underwent oxidation effect and deamination, respectively; as a result, 4-nitrobenzenesulfonic acid P6 (*m*/*z* = 204) and benzenesulfonic acid P7 (*m*/*z* = 159) were generated [100,101]. In Pathway 3, the S-C bond of the STZ was ruptured; as a result, thiazole-2-sulfonamide acid P4 (*m*/*z* = 180) and aniline P5 (*m*/*z* = 93) were produced. Subsequently, aniline P5 (*m*/*z* = 93) underwent hydroxylation; therefore, 4-aminophenol P8 (*m*/*z* = 109) and 4-aminobenzene-1,3-diol P9 (*m*/*z* = 125) were formed. Then, 4-aminophenol P8 (*m*/*z* = 109) further underwent hydroxylation for generating hydroquinone P10 (*m*/*z* = 110) [99,100,101]. The thiazole ring was attacked by the active radicals; as a result, the ring cleavage and the formation of small-molecule organic acids were realized [101,102]. Ultimately, the intermediate organic products which were generated during the photocatalytic degradation process of the STZ were further decomposed on the surface of the photocatalyst for forming the final products which included CO_2_, H_2_O, SO_4_^2−^, NH_4_^+^, and NO_3_^−^.

## 4. Conclusions

In conclusion, the BiTmFeSbO_7_ photocatalyst was successfully synthesized by using the solvothermal method for the first time. Meanwhile, the direct Z-scheme BTBTHP was successfully synthesized by means of the ultrasonic-assisted solvothermal method. A Z-scheme heterojunction structure was established between the BiTmFeSbO_7_ photocatalyst and the BiTmO_3_ photocatalyst. The BTBTHP which possessed the heterojunction structure significantly enhanced the separation efficiency of the PEPH; concurrently, the BTBTHP maintained a higher redox potential. During the photocatalytic degradation process of the STZ, the BTBTHP demonstrated excellent photocatalytic activity compared with BiTmFeSbO_7_, BiTmO_3_, and N-T. Under the condition of VLIRN, the degradation efficiency of the STZ reached as high as 99.5% by using the BTBTHP. Specifically, the degradation rate of the STZ by using the BTBTHP was 1.14 times that of using BiTmFeSbO_7_, 1.28 times that of using BiTmO_3_, and 2.71 times that of using N-T. Furthermore, the stability and reusability of the BTBTHP were verified through consecutive cycle degradation experiments. The radical trapping experiments and the EPR analysis results confirmed that •O_2_^−^, •OH, and h^+^ were involved in the efficient degradation of the STZ and played a pivotal role during the degradation process of the STZ. In addition, three credible degradation pathways of the STZ were proposed in this study. In summary, the BTBTHP possessed great potential for the treatment of antibiotic-contaminated wastewater. This study provided an effective strategy for treating antibiotic-contaminated wastewater and offered valuable insights into the development of the effective Z-scheme heterojunction photocatalysts.

## Figures and Tables

**Table 1:** The comparison results for the photocatalytic activity of the BTBTHP with other reported photocatalysts during the photodegradation process of the STZ under the condition of VLIRN.

Photocatalyst	Incident Light	Irradiation Time (min)	Name of Antibiotic	Removal Rate (%)	Reference
HTiO_2_-NS	Visible light	360	Sulfathiazole	70	[77]
C_70_-TiO_2_	Visible light	180	Sulfathiazole	90	[78]
DWCNT-N/TiO_2_	Visible light	180	Sulfathiazole	77	[79]
CN-0.1Ni/NiOx	Visible light	240	Sulfathiazole	78.5	[80]
YAG:Ce/ZnO 1/1 CSN	Visible light	300	Sulfathiazole	80	[81]
BiTmFeSbO_7_	Visible light	120	Sulfathiazole	86.91	This study
BTBTHP	Visible light	120	Sulfathiazole	99.50	This study

## Data Availability

The original contributions presented in our study are included in the article/Supplementary Materials. Further inquiries can be directed to the corresponding author(s).

## Supplementary Materials

The following supporting information can be downloaded at https://www.mdpi.com/article/10.3390/nano15231756/s1, Figure S1: The XRD pattern of the BiTmO_3_ photocatalyst; Figure S2: The XRD pattern of the BiTmFeSbO_7_ photocatalyst; Figure S3: (a) The XRD pattern and the Rietveld refinement result of the BiTmFeSbO_7_ photocatalyst and (b) the atomic structure (red atom: O, purple atom: Bi or Tm, light purple atom: Fe or Sb) of the BiTmFeSbO_7_ photocatalyst; Figure S4: (a) The XRD pattern and the Rietveld refinement result of the BiTmO_3_ photocatalyst and (b) the atomic structure (red atom: O, purple atom: Bi or Tm) of the BiTmO_3_ photocatalyst; Figure S5: The XRD spectrum of the N-doped TiO_2_ photocatalyst; Figure S6: The Raman spectrum of the BiTmO_3_ photocatalyst; Figure S7: The Raman spectrum of the BiTmFeSbO_7_ photocatalyst; Figure S8: The Raman spectrum of the BTBTHP; Figure S9: The TEM morphology image of the BTBTHP; Figure S10: The HRTEM image of the BTBTHP; Figure S11: The XRD spectrum of the BTBTHP after quintic cyclic degradation process of the sulfathiazole by using the BTBTHP under the condition of visible light irradiation; Figure S12: The ultraviolet and visible absorption spectrum of the BTBTHP after quintic cyclic degradation process of the sulfathiazole by using the BTBTHP under the condition of visible light irradiation; Figure S13: The SEM morphology pattern of the BTBTHP after quintic cyclic degradation process of the sulfathiazole by using the BTBTHP under the condition of visible light irradiation; Figure S14: The EDS element scanning mapping of the BTBTHP after quintic cyclic degradation process of the sulfathiazole by using the BTBTHP under the condition of visible light irradiation (Bi, Tm, Fe, Sb, and O from BiTmFeSbO_7_; simultaneously, Bi, Tm, and O from BiTmO_3_); Figure S15: The effect of the BTBTHP dosage on the removal efficiency of the STZ; Figure S16: The effect of different pH values on the degradation efficiency of the STZ by using the BTBTHP under the condition of visible light irradiation; Figure S17: The effect of different anions on the degradation efficiency of the STZ by using the BTBTHP under the condition of visible light irradiation; Figure S18: The effect of the initial concentration variation in the STZ on the degradation efficiency of the STZ by using the BTBTHP under the condition of visible light irradiation; Table S1: The removal rates of the STZ by using the BiTmFeSbO_7_/BiTmO_3_ heterojunction photocatalyst with different proportions under the condition of visible light irradiation; Table S2: The atomic characteristic values of the crystal structure of the BiTmFeSbO_7_ photocatalyst; Table S3: The atomic characteristic values of the crystal structure for the BiTmO_3_ photocatalyst; Section S1: Synthesis of Nitrogen-Doped TiO_2_; Section S2: Characterization.
