# Supplementary material for: Construction and Performance Characterization of BiTmFeSbO7/BiTmO3 Heterojunction Photocatalyst and the Photocatalytic Degradation of Sulfathiazole Under Visible Light Irradiation
Source: Nanomaterials (Basel). 2025 Nov 23;15(23):1756. doi: 10.3390/nano15231756 (PMC12693651; doi:10.3390/nano15231756)
Supplement: Supplementary file 1 [file nanomaterials-15-01756-s001.zip › nanomaterials-3981028-supplementary.pdf]

Construction and Performance Characterization of $BiTmFeSbO_7/BiTmO_3$ Heterojunction Photocatalyst and the Photocatalytic Degradation of Sulfathiazole under Visible Light Irradiation

Jingfei Luan [1,2,*], Xiqi Gou [1], Ye Yao [1], Liang Hao [1] and Minghe Ma [1]

[1] School of Physics, Changchun Normal University, Changchun 130032, China; 15981009457@139.com (X.G.); yaoye1109@mails.jlu.edu.cn (Y.Y.); 19845486007@139.com(L.H.); 13251704137@139.com (M.M.)
[2] State Key Laboratory of Pollution Control and Resource Reuse, School of the Environment, Nanjing University, Nanjing 210093, China
* Correspondence: jfluan@nju.edu.cn; Tel.: +86-199-5193-9498

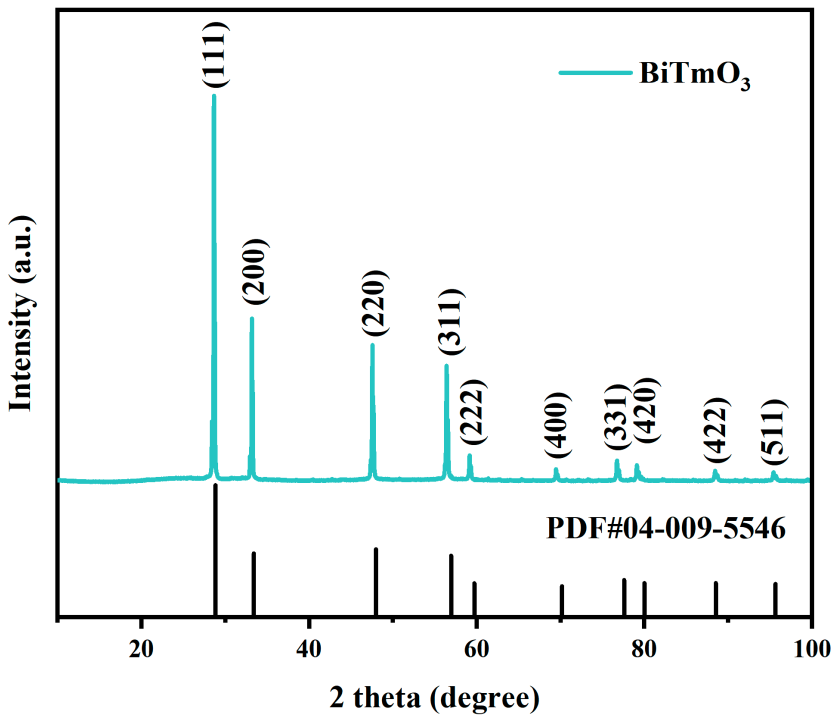

Figure S1. The XRD pattern of the $BiTmO_3$ photocatalyst.

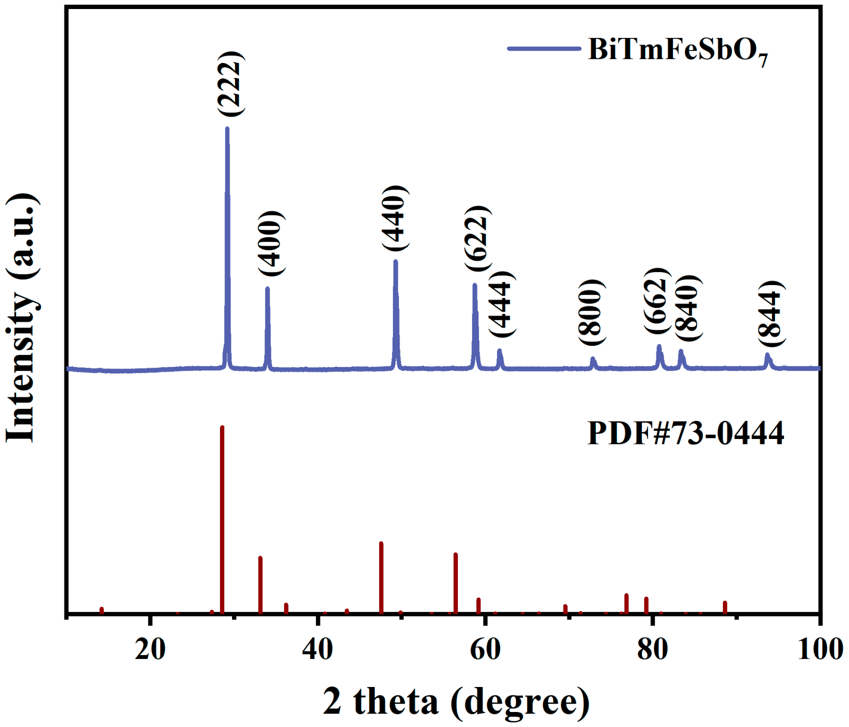

Figure S2. The XRD pattern of the $BiTmFeSbO_7$ photocatalyst.

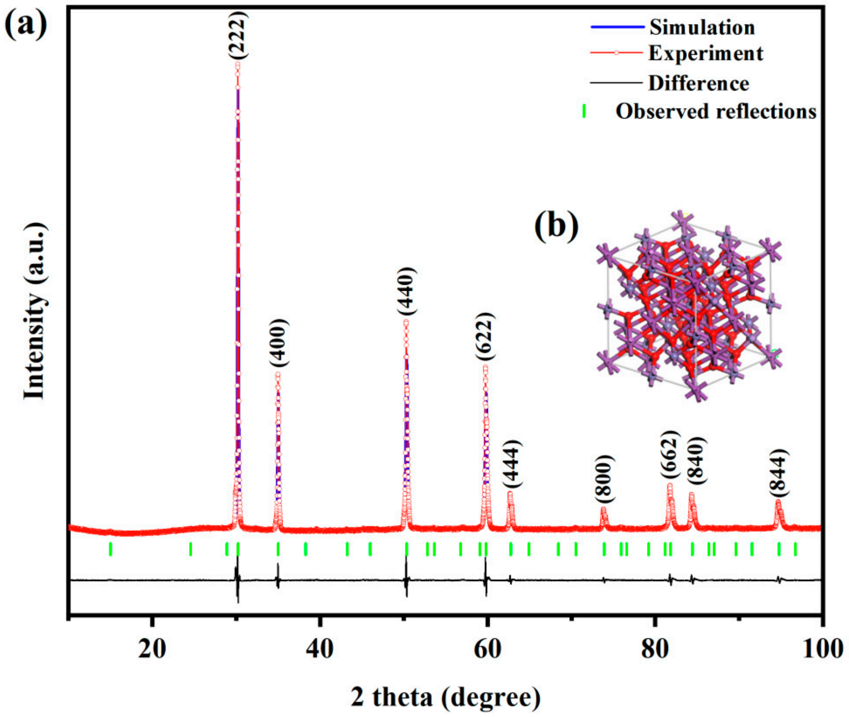

Figure S3. (a) The XRD pattern and the Rietveld refinement result of the $BiTmFeSbO_7$ photocatalyst; (b) The atomic structure (Red atom: O, purple atom: Bi or Tm, light purple atom: Fe or Sb) of the $BiTmFeSbO_7$ photocatalyst.

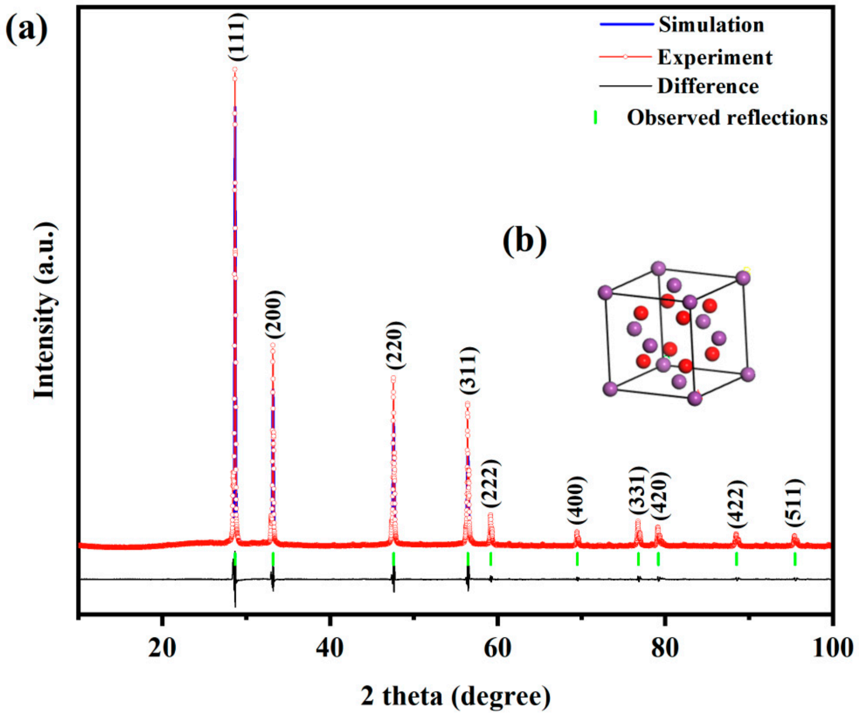

Figure S4. (a) The XRD pattern and the Rietveld refinement result of the $BiTmO_3$ photocatalyst; (b) The atomic structure (Red atom: O, purple atom: Bi or Tm) of the $BiTmO_3$ photocatalyst.

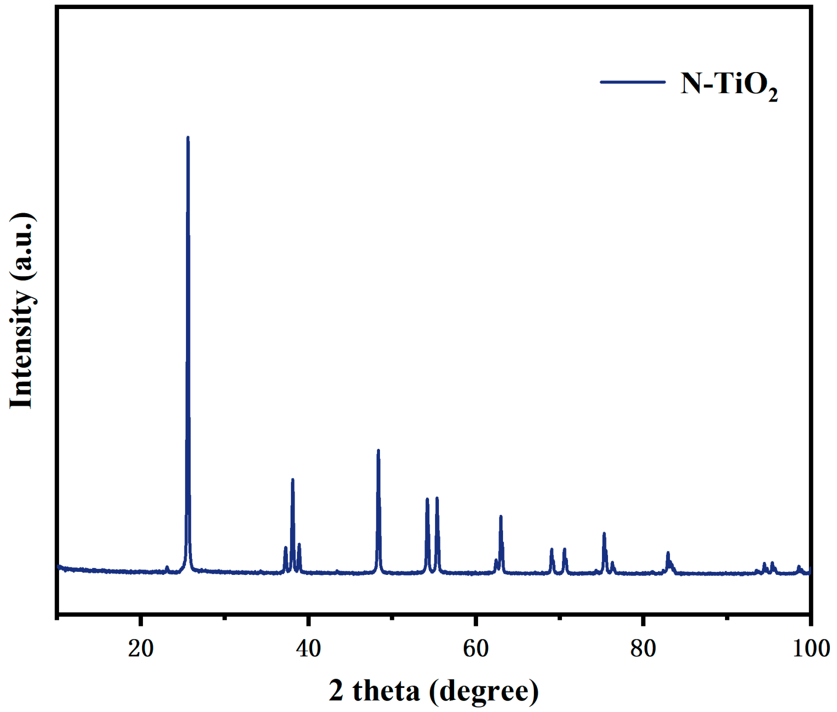

Figure S5. The XRD spectrum of the N-doped TiO_2 photocatalyst.

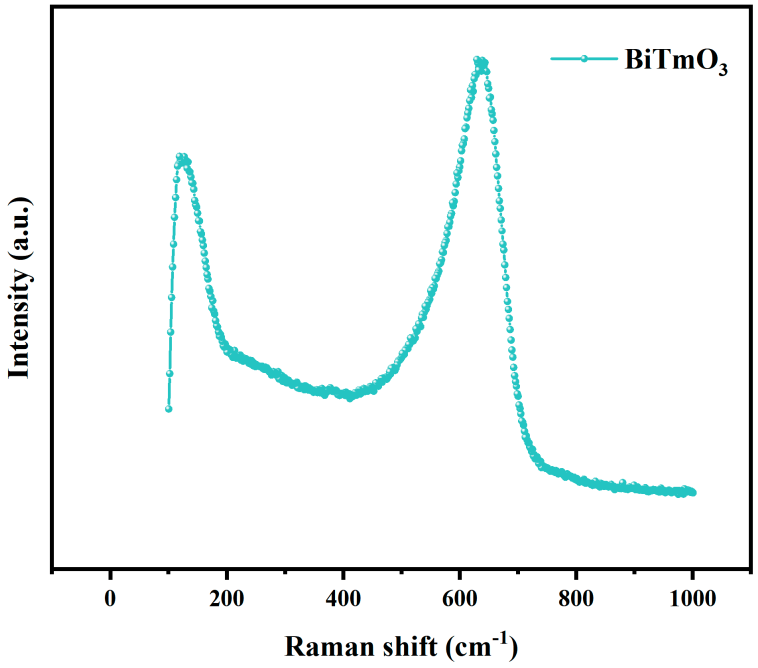

Figure S6. The Raman spectrum of the $BiTmO_3$ photocatalyst.

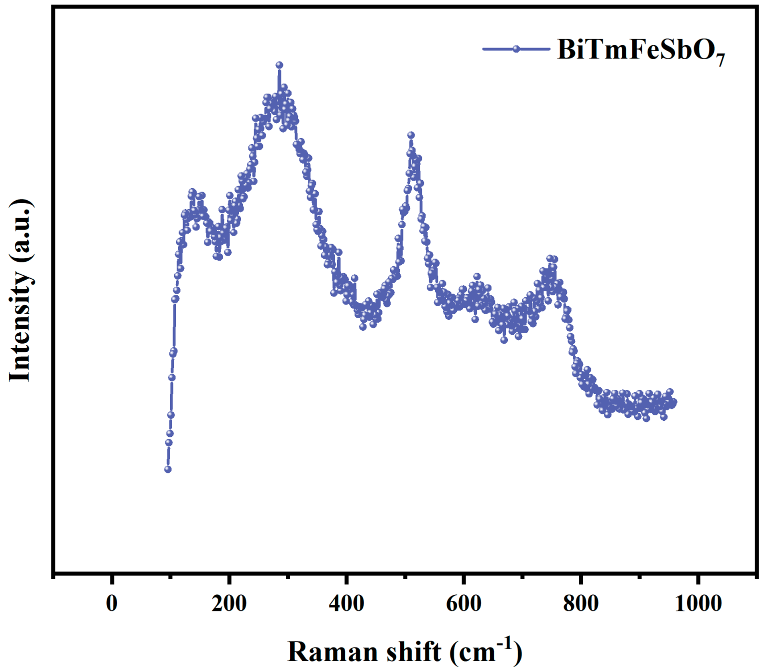

Figure S7. The Raman spectrum of the $BiTmFeSbO_7$ photocatalyst.

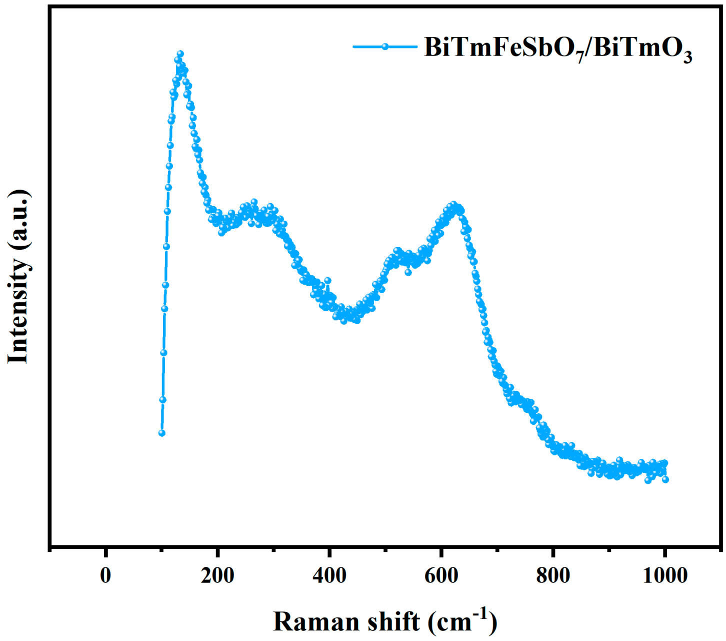

Figure S8. The Raman spectrum of the BTBTHP photocatalyst.

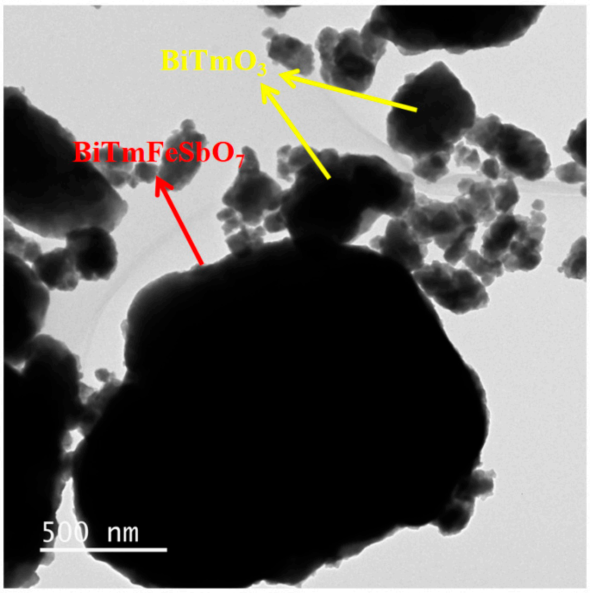

Figure S9. The TEM morphology image of the BTBTHP.

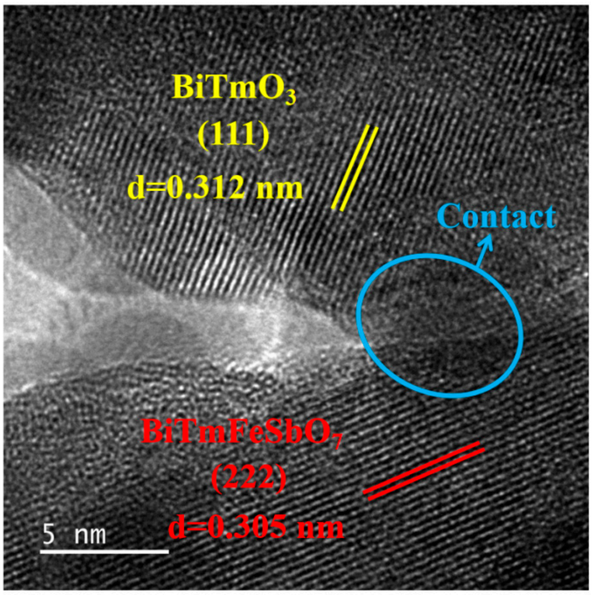

Figure S10. The HRTEM image of the BTBTHP.

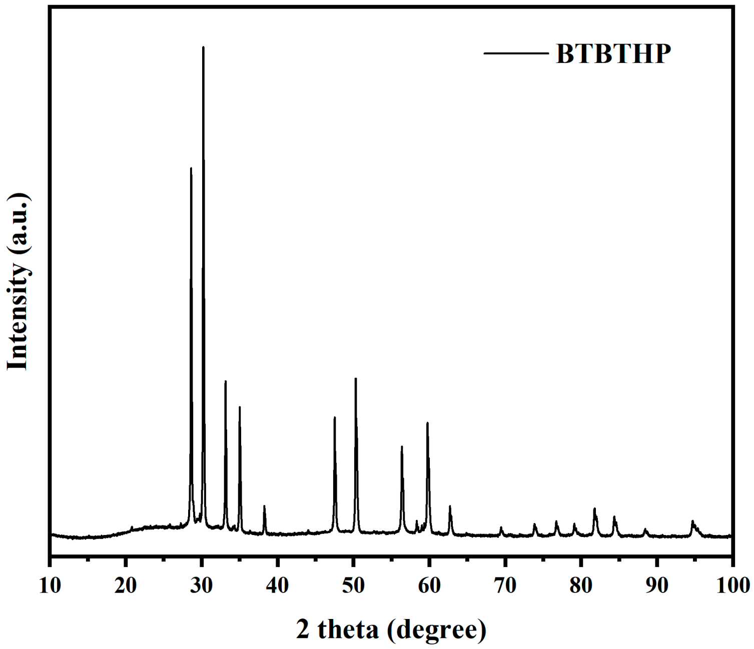

Figure S11. The XRD spectrum of the BTBTHP after quintic cyclic degradation process of the sulfathiazole by using the BTBTHP under the condition of visible light irradiation.

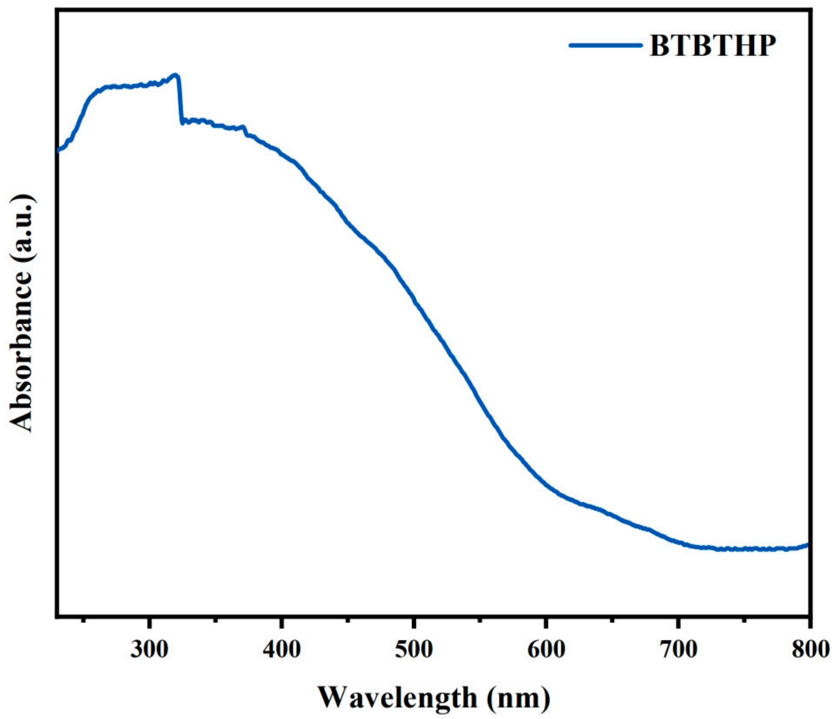

Figure S12. The ultraviolet and visible absorption spectrum of the BTBTHP after quintic cyclic degradation process of the sulfathiazole by using the BTBTHP under the condition of visible light irradiation.

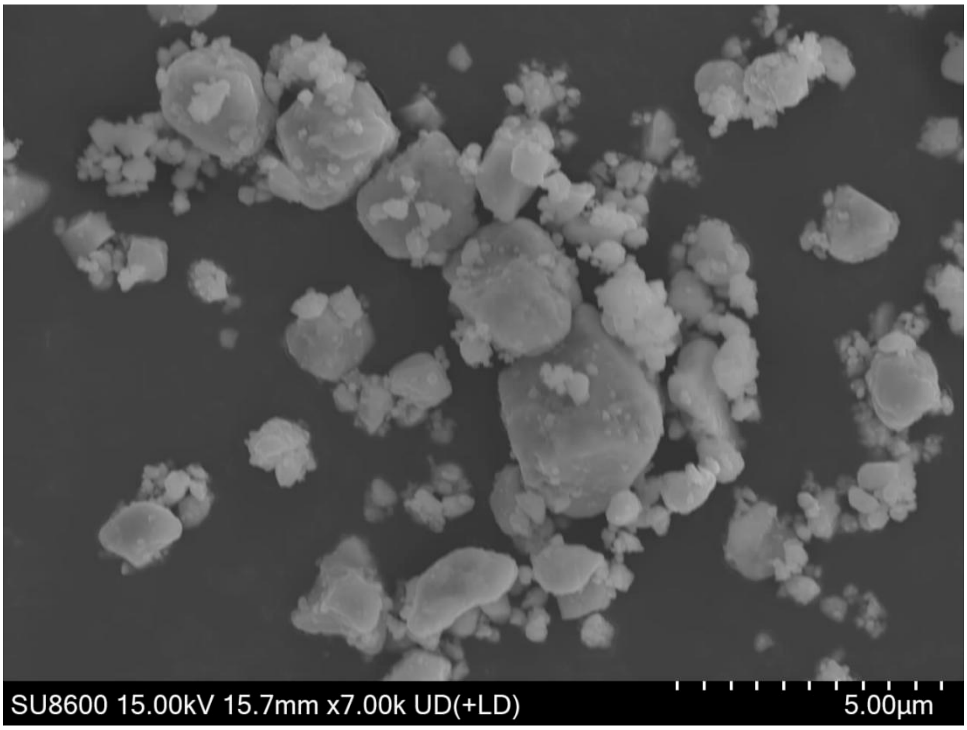

Figure S13. The SEM morphology patterm of the BTBTHP after quintic cyclic degradation process of the sulfathiazole by using the BTBTHP under the condition of visible light irradiation.

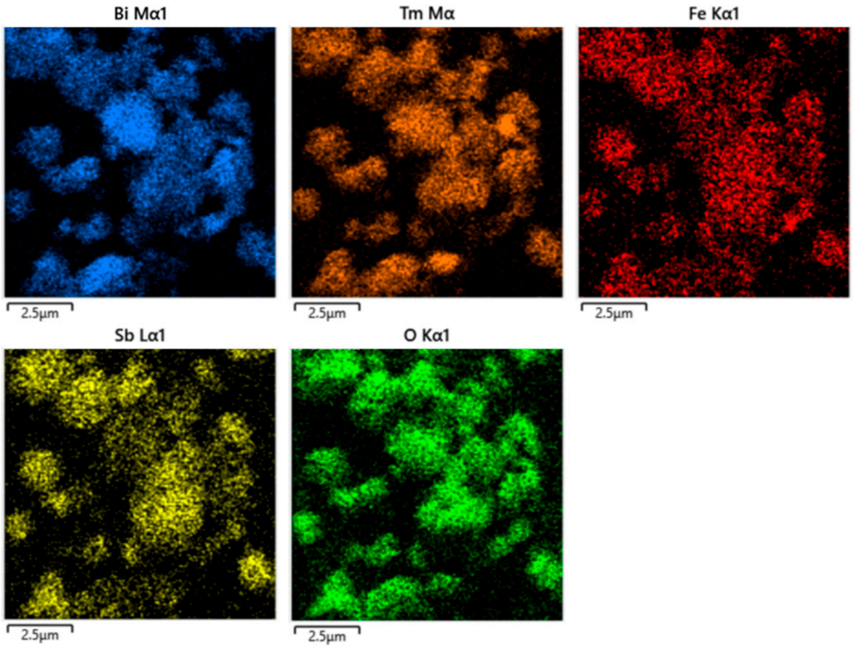

Figure S14. The EDS element scanning mapping of the BTBTHP after quintic cyclic degradation process of the sulfathiazole by using the BTBTHP under the condition of visible light irradiation (Bi, Tm, Fe, Sb, and O from $BiTmFeSbO_7$, simultaneously, Bi, Tm, and O from $BiTmO_3$).

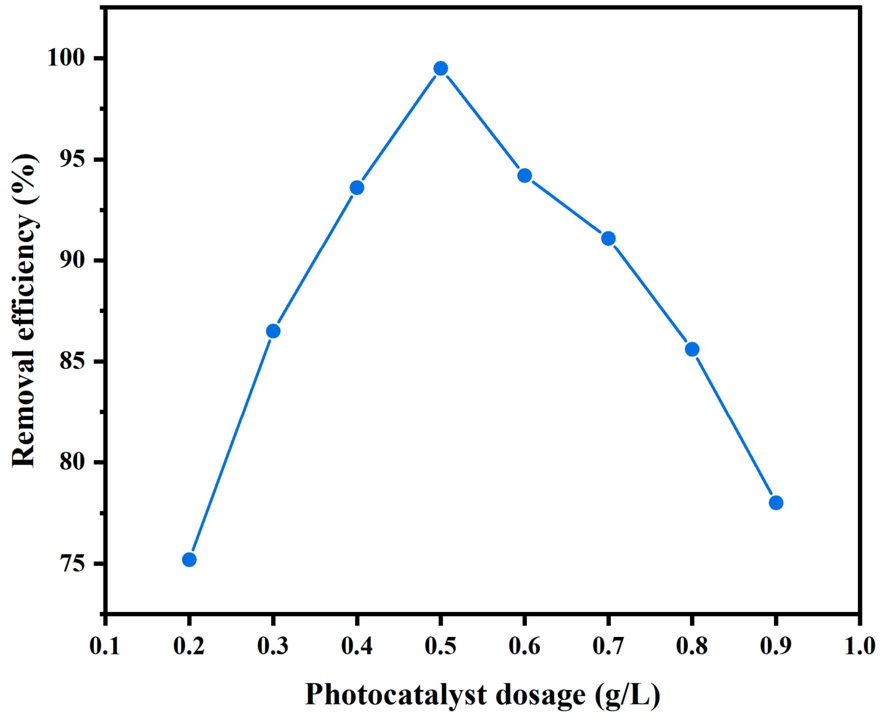

Figure S15. Effect of the BTBTHP dosage on the removal efficiency of the STZ.

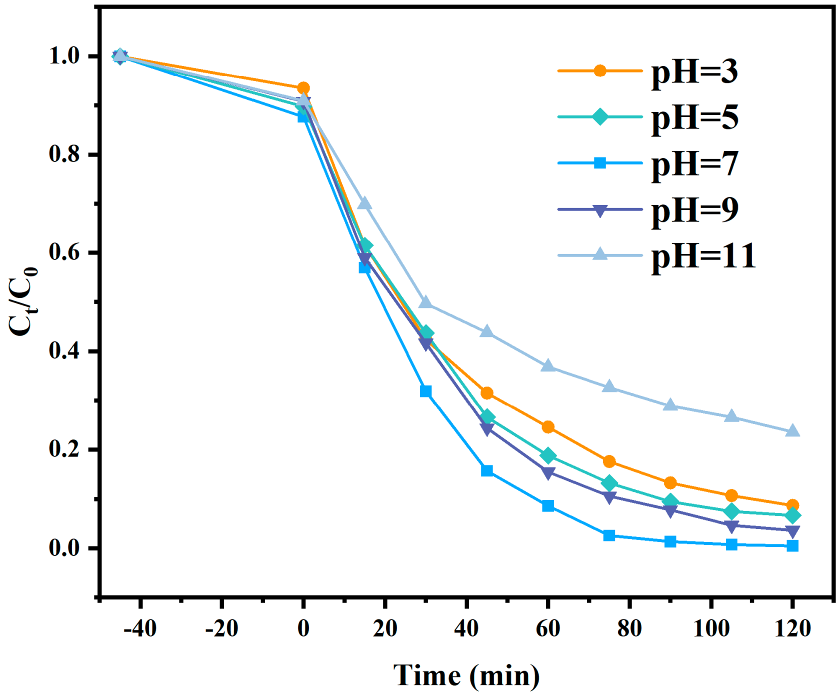

Figure S16. The effect of different pH values on the degradation efficiency of the STZ by using the BTBTHP under the condition of visible light irradiation.

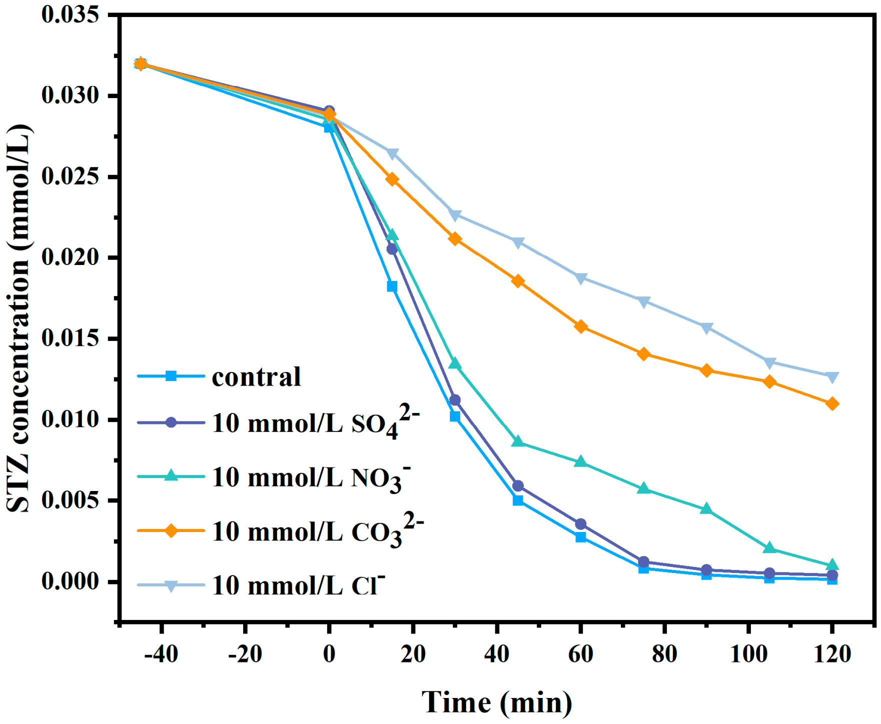

Figure S17. The effect of different anions on the degradation efficiency of the STZ by using the BTBTHP under the condition of visible light irradiation.

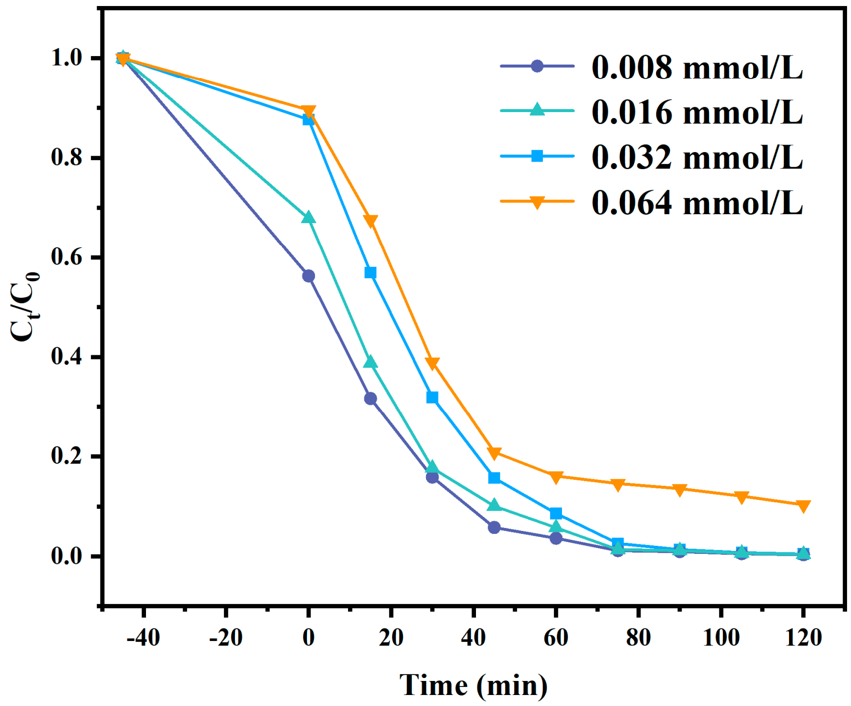

Figure S18. The effect of the initial concentration variation of the STZ on the degradation efficiency of the STZ by using the BTBTHP under the condition of visible light irradiation.

Table S1. The removal rates of the STZ by using the $BiTmFeSbO_7/BiTmO_3$ heterojunction photocatalyst with different proportions under the condition of visible light irradiation.

Photocatalyst with different proportion	Visible light irradiation time (min)	Removal efficiency of the STZ (%)
BTBTHP-(1:3)	120	74.5
BTBTHP-(1:2)	120	86.8
BTBTHP	120	99.5
BTBTHP-(2:1)	120	81.7
BTBTHP-(3:1)	120	75.3

Table S2. The atomic characteristic values of the crystal structure of the $BiTmFeSbO_7$ photocatalyst.

**Atom**	**x**	**y**	**z**	**Occupation factor**
Bi	0	0	0	0.5
Tm	0	0	0	0.5
Fe	0.5	0.5	0.5	0.5
Sb	0.5	0.5	0.5	0.5
O(1)	-0.175	0.125	0.125	1
O(2)	0.125	0.125	0.125	1

Table S3. The atomic characteristic values of the crystal structure for the $BiTmO_3$ photocatalyst.

**Atom**	**x**	**y**	**z**	**Occupation factor**
Bi	0	0	0	0.5
Tm	0	0	0	0.5
O	0.25	0.25	0.25	0.75

Section S1. Synthesis of Nitrogen-Doped TiO_2

In this study, the N-doped TiO_2 (N-T) was prepared by using the sol-gel method. Initially, a specific quantity of tetrabutyl titanate was blent with absolute ethanol for forming solution A, and then, the glacial acetic acid, the double-distilled water, and the absolute ethanol were mixed for forming solution B. The tetrabutyl titanate could be utilized as the precursor, meanwhile, the ethanol was used as the solvent. Under the condition of magnetic stirring, the solution A and the solution B were mixed for 30 minutes, as a result, a translucent gel-like suspension was obtained. Subsequently, different amounts for 1 mol/L ammonia solution were incrementally introduced into the suspension for maintaining the molar ratio of N/Ti at 8%. The stirring operation was continued for 60 minutes, and then the gel was air-dried at room temperature for 48 hours, therefore, a solid gel was formed. The dried gel was comminuted and calcined at 500°C for 3 hours. In the end, a vibrating sieve was utilized for gaining the titanium dioxide catalyst which was doped with nitrogen.

Section S2. Characterization

The X-ray diffraction (XRD) analysis was conducted using an XRD-6000 (Shimadzu Corporation, Kyoto, Japan) to obtain crystal structure information. The Fourier-transform infrared spectroscopy (FTIR) analysis was performed with a WQF-530A (Beijing North Finray Analytical Instrument (Group) Co., Ltd., Beijing, China) to identify functional groups and chemical bonds. The Raman spectroscopy (INVIA0919-06, RENISHAW plx, Walton-under-edge, London, UK) was utilized to examine the interactions among different chemical bonds. The ultraviolet-visible diffuse reflectance spectrophotometry (UV-Vis DRS) analysis was conducted using a UV-3600 (Shimadzu Corporation, Kyoto, Japan) to analyze the band gap and optical properties of the samples. The X-ray photoelectron spectroscopy (XPS) was conducted using a PHI 5000 (VersaProbe, UlVAC-PHI, Chigasaki, Japan) to analyze the surface chemical composition and oxidation states. The transmission electron microscopy (Talos F200X G2, TEM, Thermo Fisher Scientific, Waltham, MA, USA) was employed to analyze the microstructure and morphological features of the samples, and the energy-dispersive spectroscopy (EDS) was used to determine the constituent elements. The ultraviolet photoelectron spectroscopy (UPS) was conducted using an Escalab 250 xi (Thermo Fisher Scientific, Waltham, MA, USA) to measure the ionization potential of the valence band. The FLS1000 fluorescence spectrom-eter (Edinburgh Instruments, Edinburgh, UK) was used to measure the photoluminescence (PL) properties and fluorescence lifetime of the catalysts. The electron paramagnetic resonance (EPR) spectroscopy was conducted using an A300 instrument(Bruker Corporation, Karlsruhe, Germany) to detect free radicals in the samples. The photocurrent spectroscopy analysis was performed with a CS310H (Wuhan Coster Instrument Co., LTD, Wuhan, China) to compare the photocurrent response intensities of the samples.
